# A Multi-Objective Sine Cosine Algorithm Based on a Competitive Mechanism and Its Application in Engineering Design Problems

**DOI:** 10.3390/biomimetics9020115

**Published:** 2024-02-15

**Authors:** Nengxian Liu, Jeng-Shyang Pan, Genggeng Liu, Mingjian Fu, Yanyan Kong, Pei Hu

**Affiliations:** 1 College of Computer and Data Science, Fuzhou University, Fuzhou 350108, China; lylnx@fzu.edu.cn (N.L.); liugenggeng@fzu.edu.cn (G.L.); sinceway@fzu.edu.cn (M.F.); 2 School of Artificial Intelligence, Nanjing University of Information Science & Technology, Nanjing 210044, China; jspan@cc.kuas.edu.tw; 3 School of Information Science and Engineering, ZheJiang Sci-Tech University, Hangzhou 310018, China; 4 School of Computer and Software, Nanyang Institute of Technology, Nanyang 473004, China

**Keywords:** multi-objective algorithm, sine cosine algorithm (SCA), competitive mechanism, engineering design problem

## Abstract

There are a lot of multi-objective optimization problems (MOPs) in the real world, and many multi-objective evolutionary algorithms (MOEAs) have been presented to solve MOPs. However, obtaining non-dominated solutions that trade off convergence and diversity remains a major challenge for a MOEA. To solve this problem, this paper designs an efficient multi-objective sine cosine algorithm based on a competitive mechanism (CMOSCA). In the CMOSCA, the ranking relies on non-dominated sorting, and the crowding distance rank is utilized to choose the outstanding agents, which are employed to guide the evolution of the SCA. Furthermore, a competitive mechanism stemming from the shift-based density estimation approach is adopted to devise a new position updating operator for creating offspring agents. In each competition, two agents are randomly selected from the outstanding agents, and the winner of the competition is integrated into the position update scheme of the SCA. The performance of our proposed CMOSCA was first verified on three benchmark suites (i.e., DTLZ, WFG, and ZDT) with diversity characteristics and compared with several MOEAs. The experimental results indicated that the CMOSCA can obtain a Pareto-optimal front with better convergence and diversity. Finally, the CMOSCA was applied to deal with several engineering design problems taken from the literature, and the statistical results demonstrated that the CMOSCA is an efficient and effective approach for engineering design problems.

## 1. Introduction

Multi-objective optimization problems (MOPs) arise from engineering applications [1], community detection problems [2], charging station placement problems [3], the detection of ice accretion on aircraft [4], etc. MOPs often involve multiple conflicting objectives [5,6], such as environmental/economic dispatch (EED) problems with the two objectives of minimizing pollution emissions and generation costs, and there does not exist a solution that is able to optimize all objectives simultaneously. The mathematical model of the MOP for minimization is formulated as follows:

(1) $$\begin{array}{cc} \min & F\left(x\right)=(f_{1}\left(x\right),f_{2}\left(x\right),\ldots ,f_{m}\left(x\right))\\ s.t. & g_{i}\left(x\right)\leq 0\;\;\;\;\left(p=1,2,\ldots ,p\right)\\ & h_{j}\left(x\right)=0\;\;\;\;\left(q=1,2,\ldots ,q\right) \end{array}$$

where $x=(x_{1},x_{2},\ldots ,x_{D})$ denotes the *D*-dimensional candidate solution in decision space; $f_{1}\left(x\right)$, $f_{2}\left(x\right)$, …, $f_{m}\left(x\right)$ are objective functions; and *m* is the number of objectives. $g_{i}\left(x\right)$ and $h_{j}\left(x\right)$ are constraint functions. Given two candidate solutions $x_{a}$ and $x_{b}$ in the feasible region, $x_{a}$ dominates $x_{b}$, if and only if ∀i, $f_{i}\left(x_{a}\right)\leq f_{i}\left(x_{b}\right)$ and ∃j, $f_{j}\left(x_{a}\right)<f_{j}\left(x_{b}\right)$, i,j∈{1,2,…,m}. If no other solution can dominate $x^{*}$, then $x^{*}$ is called the Pareto-optimal solution. The set of all Pareto-optimal solutions is known as the Pareto-optimal set (PS), and the set of their corresponding objective values is called the Pareto front (PF).

In the past twenty years, multi-objective evolutionary algorithms (MOEAs) have achieved great popularity due to their excellent capabilities in dealing with MOPs [7,8]. In 1985, Schaffer introduced the first MOEA, i.e., the vector-evaluated genetic algorithm (VEGA) [9]. After the VEGA, a large number of MOEAs based on various metaheuristic algorithms were presented, such as the whale optimization algorithm [10], particle swarm optimization (PSO) [11], the carnivorous plant algorithm [12], the equilibrium optimizer slime mould algorithm [1], differential evolution (DE) [13,14], the butterfly optimization algorithm [15], the remora optimization algorithm [16,17], the crayfish optimization algorithm [18], and the gray wolf optimizer [19]. Based on their evolution schemes, MOEAs can be roughly partitioned into three types.

The first type is dominance-based MOEAs. These rank solutions according to the dominance relationship and choose solutions for the next generation based on Pareto and diversity selection criteria. The most representative and famous example for MOPs is the fast elite multi-objective GA (NSGA-II), which was introduced by Deb et al. [20]. To further address Many-objective optimization problems, scholars designed several new dominance rules, such as θ-dominance [21], grid dominance [22], fuzzy dominance [23], and ϵ-dominance [24]. Recently, inspired by the idea of a competitive swarm optimizer (CSO) [25], Zhang et al. developed a multi-objective PSO (CMOPSO) that utilizes a pairwise competitive mechanism to update the velocity of particles [26]. The comparison results demonstrated that the CMOPSO performed better than the competing algorithms. To enhance the performance of the CMOPSO, Han et al. adopted a tripartite competition mechanism to propose an improved multi-objective PSO, called the TC-MOPSO [27]. Using the level swarm optimizer and a competition mechanism, Zhang et al. introduced an MOEA named EMOSO [28].

The second type is decomposition-based MOEAs. This type can be further classified into two categories, one wherein the MOP is transformed into a series of single-objective sub-problems [29,30], and the other wherein a complex MOP is transformed into a series of simple MOPs [31]. A representative example of a decomposition-based MOEA is the MOEA/D [29]. The same authors introduced a new variant of the MOEA/D with DE operators, called MOEA/D-DE [32]. What is more, some advanced versions of the MOEA/D have been proposed to handle more challenging MOPs, such as MOEA/D-PaS [33] and MOEA/D-AM2M [34]. Using a decomposition strategy, Cheng et al. presented an MOEA with a set of reference vectors, named the RVEA [35]. Furthermore, Zhao et al. introduced a surrogate-ensemble assisted MOEA on the basis of the RVEA to deal with expensive problems [36]. Recently, Yang et al. introduced an MOEA with a dual decomposition strategy to solve large-scale multi-objective optimization problems [37].

The third type is indicator-based MOEAs, which utilize performance indicators rather than fitness for evolutionary selection. Beume et al. [38] introduced an s-metric selection-based MOEA, called the SMS-EMOA, via embedding the hypervolume (HV) with a non-dominated sorting design. Zitzler et al. developed an indicator-based evolutionary algorithm (IBEA), in which the HV and ε-indicator are employed in the evolutionary selection [39]. However, the computational time for the HV rapidly increases as the number of objectives increases. Hence, Bader et al. developed a hypervolume-based MOEA (Hype), which adopts Monte Carlo simulations to calculate the HV values [40]. To enhance the performance of MOEAs for irregular Pareto frontier problems, Tian et al. presented an indicator-based MOEA (AR-MOEA) whose reference points are adaptive [41].

Additionally, many researchers have combined the above three strategies and proposed some efficient MOEAs. Li et al. developed an MOEA/DD method using both decomposition and dominance mechanisms [42]. Based on the famous NSGA-II, Deb et al. proposed an improved MOEA (NSGA-III) on the basis of a decomposition mechanism and non-dominated sorting strategy [43]. Wang et al. presented a MOEA (Two-arch2) by utilizing both the dominance and the performance indicator [44]. Although researchers have proposed many MOEAs to improve the convergence performance and maintain diversity and attained better performance, the balance of convergence and diversity is still a major challenge in multi-objective optimization.

In this paper, to promote the effectiveness of the MOEA in addressing MOPs, an efficient multi-objective sine cosine algorithm using an SDE-based competitive mechanism is proposed. The competitive idea utilized in the CSO is an effective mechanism that has also been employed to enhance the performance of other methods [10,26,45,46]. The sine cosine algorithm (SCA) is an effective meta-heuristic introduced in 2016 [47]. The SCA has demonstrated its robustness and effectiveness in terms of accuracy, convergence, and computational efforts [48]. However, the SCA has not yet been integrated with competitive mechanisms for handling MOPs. To take advantages of the SCA and the effectiveness of the competitive mechanism, we develop a multi-objective SCA based on the competitive mechanism, named the CMOSCA. The contributions of this work are summarized as follows.

- A new position updating operation based on a competitive mechanism with the shift-based density estimation (SDE) strategy is proposed. In this operation, an agent with better SDE fitness value is employed to guide the search of evolution. This operation can make use of the SDE-based competitive mechanism to attain a well balance between the diversity and convergence.
- We also present two variants of the CMOSCA, which utilize the Euclidean distance-based competitive mechanism and angle-based competitive mechanism, respectively. The performance of these two variants with CMOSCA was experimentally compared, and the experimental results indicate the virtue of the SDE-based competition mechanism.
- The performance of the CMOSCA is extensively analyzed via comparing CMOSCA with several representative MOEAs on twenty test functions having various landscapes of Pareto fronts. Furthermore, the proposed CMOSCA is also applied to address several engineering design problems. The comparison results evidence the competitive performance of our proposed CMOSCA.

The remaining article is organized as follows. Section 2 describes the idea of the SCA and related works on multi-objective SCA. Section 3 provides our proposed CMOSCA in detail. Experiments on twenty test functions and several engineering design problems with some discussions are given in Section 4. At last, the conclusions are summarized in Section 5.

## 2. Related Work

### 2.1. Sine Cosine Algorithm (SCA)

The SCA is a population-based meta-heuristic optimization approach, proposed by Seyedali Mirjalili in 2016, which is inspired by the mathematical characteristics of sine and cosine functions [47]. It employs the rules of trigonometric sine and cosine functions to update the positions of agents for the optimal solution. The positions of agents in the SCA are updated utilizing the Equation (2).

(2) $$\left\{\begin{array}{c} x_{i}^{t+1}=x_{i}^{t}+r_{1}\;\times \;\sin\left(r_{2}\right)\;\times \;\left|r_{3}p_{i}^{t}-x_{i}^{t}\right|\;\;r_{4}<0.5\\ x_{i}^{t+1}=x_{i}^{t}+r_{1}\;\times \;\cos\left(r_{2}\right)\;\times \;\left|r_{3}p_{i}^{t}-x_{i}^{t}\right|\;\;r_{4}\geq 0.5 \end{array}\right.$$

where $x_{i}^{t+1}$, $x_{i}^{t}$ are the *i*th (i=1,2,…,N) agents at (t+1)th and *t*th iteration respectively and $p_{i}^{t}$ represents the best agent (destination point) at the *t*th iteration, and $r_{1},r_{2},r_{3}$, and $r_{4}$ are random control parameters. These parameters are integrated to prevent the algorithm from getting stuck in local optima and to balance the exploration and exploitation of the search process.

The parameter $r_{1}$ makes a contribution to the exploration in the first half of the iterative search process and to the exploitation in the second half of the iterative search process. To balance exploration and exploitation, $r_{1}$ decreases adaptively from a preset constant *a* to 0 with the following linear equation.

(3) $$r_{1}=a-t\frac{a}{T}$$

where *a* is a constant, *t* and *T* represent the current and maximum iteration, respectively.

**Figure 1:**
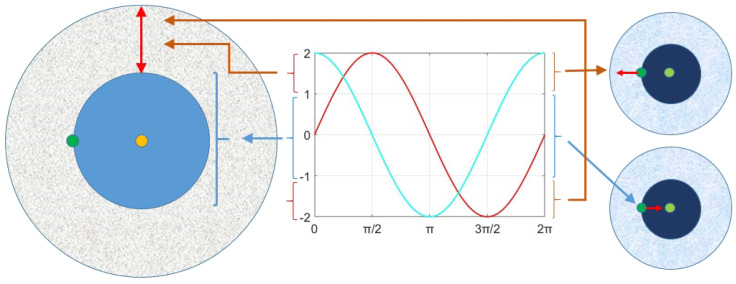
The influence of sine and cosine functions in Equation (2) on the next position in the [−2,2] interval.

The $r_{2}$ is a direction parameter, which indicates the movement of the current agent either toward or outside of the destination. $r_{2}$ is in the range of [0,2Π]. The $r_{3}$ is a weight parameter, which is used to randomly emphasize ($r_{3}>1$) or weaken ($r_{3}<1$) the impact of the destination on other solution agents. The range of $r_{3}$ is [0,2]. Finally, $r_{4}$ is a uniformly random parameter in the range of [0,1], which performs as a switch to equally select sine or cosine function in Equation (2). The influence of the sine and cosine functions in Equation (2) on the next position in the [−2,2] interval is illustrated in Figure 1.

**Figure 2:**
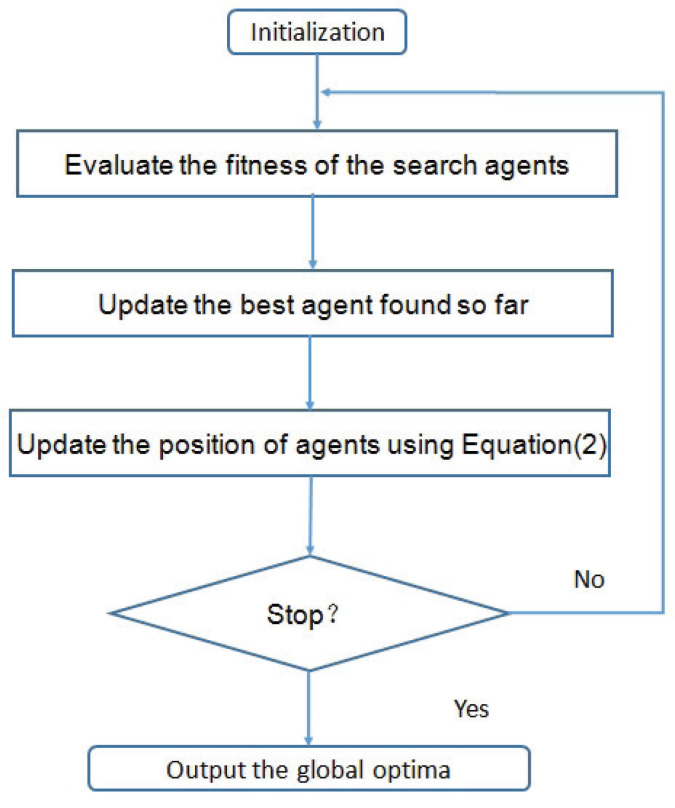
Flowchart of the Sine Cosine Algorithm.

Like other optimization approaches, The SCA begins with an initialization step, where a population of agents (initial solutions) is generated in a stochastic manner. These agents are updated iteratively via Equation (2) and the iterative process stops when the termination condition is met. The flowchart of the SCA is illustrated in Figure 2.

### 2.2. Existing Multi-Objective SCA Algorithms

In recent years, many ideas have been combined with the original SCA to propose multi-objective SCA (MOSCAs) for solving MOPs. In this part, some recently proposed multi-objective SCA methods are introduced in brief.

Rizk-Allah et al. [49] introduced a multi-objective SCA, called the MSCO, to deal with the nonsmooth EELD problem. In the MSCO, random initialization and opposition strategy are used to maintain the diversity of agents. The Pareto front concepts are employed to find a group of non-dominated solutions. The MSCO method was verified on 6-unit and 10-unit test systems, and the results evidenced the effectiveness and robustness of the MSCO.

Tawhid and Savsani presented a multi-objective SCA (MO-SCA) to deal with various benchmark MOPs and some engineering design problems [50]. In the MO-SCA, the elitist non-dominated sorting strategy and the crowding distance method are employed to define the non-dominated ranks and enhanced coverage of the obtained Pareto optimal solutions. The attained results reveal that the MO-SCA can effectively create the Pareto fronts.

Wan et al. introduced a multi-objective SCA (MOSCA) for band selecting of hyper-spectral image (HSI) [51]. The effectiveness of the MOSCA was assessed on two real-world HSI scenes and the obtained experimental results reveal the superior performance of the MOSCA compared to other competing methods. In 2022, Wan et al. further presented a multi-objective SCA for spatial-spectral clustering of remote sensing image data (MOSCA-SSC), which treats the clustering task as a multi-objective optimization problem [52]. In the MOSCA-SCC, the destination agent is automatically chosen and renewed from the current Pareto front via utilizing the knee-point-based selection method.

Abdel-Basset et al. presented a multi-objective technique to handle the task scheduling in multiprocessor systems (MPS) with the modified SCA (MSCA) to optimize both the makespan and energy objectives [53]. This algorithm uses the Pareto dominance strategy and is called energy-aware multi-objective MSCA (EA-M2SCA). Furthermore, the EA-M2SCA was hybridized with the polynomial mutation mechanism to enhance its performance and promote the convergence behavior. This hybrid improved version is called the EA-MHSCA. Finally, the proposed EA-MHSCA is compared with many well-established MOEAs, and the EA-MHSCA shows superiority in most test cases.

Wang et al. [54] developed a multi-objective SCA (MOSCA) for wind speed forecasting, in which a hybrid wavelet neutral network based on the MOSCA is proposed and experimental results show that the proposed algorithm achieved better accuracy and stability.

Selim et al. [55] introduced an efficient multi-objective SCA for the optimal allocation of distribution static compensators in distribution networks, which employs a grey relational analysis and a fuzzy loss sensitivity factor. The experimental results reveal the effectiveness and superiority of the proposed algorithm.

Altay and Alatas [56] proposed DE and SCA based novel hybrid multi-objective methods for numerical association rule mining. This study proposes three new hybrid approaches by integrating sine cosine operators into the DE method and using global exploration capability of the DE and local exploitation capability of the SCA.

Raut et al. [57] introduced Pareto multi-objective SCA for performance improvement of radial distribution network, where both the self-adapting levy mutation and exponential variation of the conversion parameter are used to improve its performance.

Narayanan et al. [58] proposed a new Many-objective SCA (MaOSCA), which uses reference points and information feedback mechanisms, and experimental results show that the MaOSCA can obtain effective and robust performance.

More related works on the MOSCAs can be found in [48,59,60,61]. Literature reviews indicate that no previous work has utilized the competitive mechanism to develop the SCA for handling multi-objective optimization problems.

In this paper, we proposed a multi-objective SCA algorithm based on a competition mechanism, which is different from previous multi-objective algorithms. For example, compared with paper [56], paper [56] proposed a hybrid multi-objective algorithm based on DE and SCA for numerical association rule mining. The proposed algorithm uses the SCA operator instead of the DE operator. Compared with paper [55], paper [55] proposed an effective multi-objective SCA by using a gray correlation analysis and fuzzy loss sensitivity factors. Among them, the gray correlation analysis is used to select leader solutions, while our proposed CMOSCA uses a competition mechanism to select leader individuals. Compared with the paper [13], which employed a competition mechanism to develop a multi-objective DE algorithm, our proposed CMOSCA utilizes a competition mechanism to design a new SCA algorithm. Compared with the paper [53], the paper [53] developed a multi-objective SCA algorithm to deal with task scheduling problems. The main innovations of this algorithm include individual initialization, individual encoding and mutation operations.

## 3. The Proposed CMOSCA

In this section, the details of our proposed CMOSCA are given. We first describe the complete framework of the CMOSCA, and then develop the position updated strategy for the SCA based on the competition mechanism with the SDE strategy. Third, two variants of our proposed CMOSCA are provided. Finally, we analyze the time complexity of the CMOSCA algorithm.

### 3.1. The Framework of the CMOSCA

**Figure 3:**
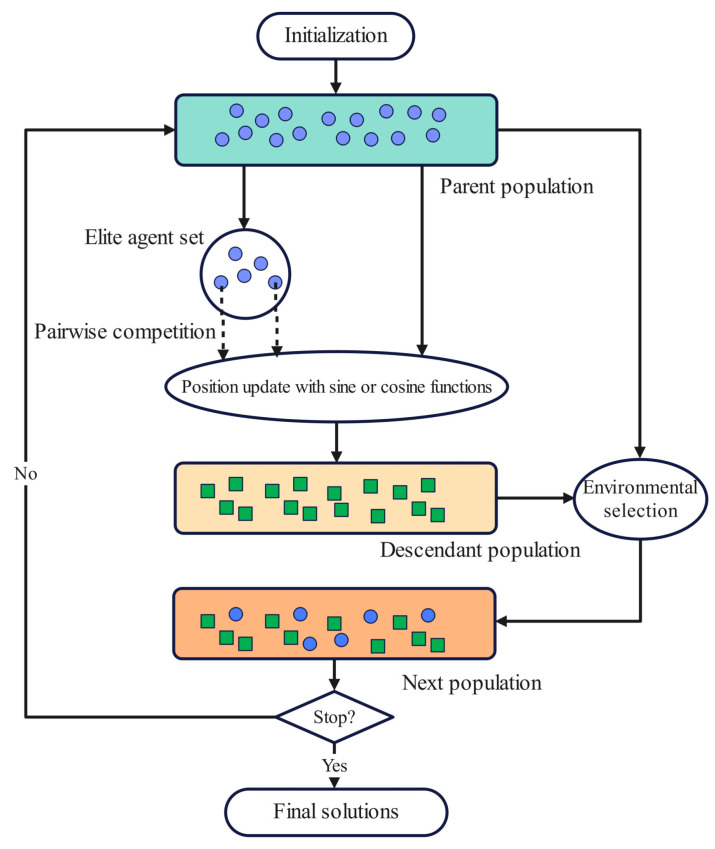
The framework of CMOSCA. 1. Elite agent set is used to guide the search of the population. 2. Pairwise competition is employed to select a winner to participate in the position update of the agent. 3. Environmental selection is adopted to choose *N* agents from the parent population and descendant population.

Similar to the CMOPSO, our proposed CMOSCA has a simple and clear framework as given in Algorithm 1, where the main loop involves two modules, namely, the competition mechanism based position updating scheme for search agents and environment selection. To illustrate more clearly, the main framework of the CMOSCA is presented in Figure 3. It is worth mentioning that this framework follows the general framework of other evolutionary algorithms, such as the GA, which uses crossover and mutation operators to produce offspring, and selects individuals from both parent and descendant populations to form a new population.
**Algorithm 1** The framework of CMOSCA**Input:** Number of search agents *N*, Dimension of the problem *D*, Maximum number of function evaluations MaxFES, Current number of function evaluations FES.**Output:** All non-dominant search agents in *P*.1:Initialize the search agents of *P*2:**while** (FES<MaxFES) **do**3:    $P^{\prime }$← CompetitionBasedSCAPositionUpdate(*P*);4:    *P*← EnvironmentalSelection(*P*,$P^{\prime }$);5:**end while**6:**Return** *P*

The process of implementing the CMOSCA is provided as follows. Firstly, the population of search agents is initialized in a random manner. Then the search agents in the population *P* are renewed by the proposed SCA position update scheme to generate descendants $P^{\prime }$. At last, we adopt the environment selection to choose *N* agents from the parent population *P* and descendant population $P^{\prime }$. Note that the CMOSCA utilizes the same environmental selection approach as the paper [62] in the iterative process. The above process (namely, SCA position updating and environmental selection) terminates when the stopping condition is met.

### 3.2. The SCA Position Update Scheme Based on a Competition Mechanism

There are four components in the SCA position update scheme with an SDE-based competition mechanism, i.e., creating the elite agent set *E*, fitness value calculation, pairwise competition, and the SCA position update. The pseudo-code of the SCA position update scheme with an SDE-based competition mechanism is presented in Algorithm 2.
**Algorithm 2** CompetitionBasedSCAPositionUpdate(*P*)**Input:** current population *P*, the size of elite agent set β.**Output:** descendant population $P^{\prime }$.1:$P^{\prime }$←Φ; /*Choosing the outstanding agents to form the elite agent set*/;2:*E*← Choose β agents from *P* relied on the front index and crowding distance.3:Fitness← Evaluate the fitness value of each agent in *E* by Equation (4);4:**for** each agent $x_{i}$ in *P* **do**5:    {a,b}← Randomly choose two agents from *E*6:    **if** Fitness(a) < Fitness(b) **then**7:         $x_{l}=a;$ //loser in the pairwise competition8:         $x_{w}=b;$ //winner in the pairwise competition9:    **else**10:         $x_{l}=b;$11:         $x_{w}=a;$12:    **end if**13:    Update $r_{1}$, $r_{2}$, and $r_{4}$, randomly;14:    Generate new $x_{i}^{\prime }$ by Equation (5);15:    $P^{\prime }$←$P^{\prime }⋃\left\{x_{i}^{\prime }\right\}$;16:**end for**17:**Return**  $P^{\prime }$

First, the elite agent set is generated (line 2 of Algorithm 2). The elite agent set plays a major role in the proposed SCA position update scheme because it provides candidate agents employed in pairwise competition to lead the search of the population. Similar to the CMOPSO, the elite agent set utilizes the approach proposed in the NSGA-II [20] to select elite agents, i.e., a non-dominated sorting and crowding distance based ranking approach which can maintain better diversity and convergence. Specifically, the non-dominated sorting is first conducted on the population *P* to get the Pareto fronts $F_{1},F_{2},\ldots ,F_{p}$, where *p* represents the maximum index of fronts. Then, the minimum number *q* is found such that $|F_{1}\cup F_{2}\cup \cdots \cup F_{q}|\geq \beta$, where β denotes the number of elite agents to be chosen. Finally, all agents belonging to first q−1 fronts are chosen as the elite agents and the remaining agents are chosen from $F_{q}$ based on the crowding distance of each agent in $F_{q}$. It is worth mentioning that the search agents in the elite agent set are chosen directly from the current population, so the CMOSCA does not require an external archive to record non-dominated solutions. Additionally, the elite agent size β has a crucial impact on the effectiveness of the CMOSCA, and we will provide a detailed discussion on β in the experimental section.

Then, we utilize the shift-based density estimation (SDE) method [63] to evaluate the fitness value of each agent in the elite agent set *E* (line 3 of Algorithm 2). To be Specifical, the minimum SDE-based distance Equation (4) [46] is adopted to evaluate the fitness value of an agent *x*.

(4) $$Fitness\left(x\right)=\min_{y\in P\setminus \{x\}}\sqrt{\sum_{i=1}^{m}{\left(\max\left\{0,f_{i}\left(y\right)-f_{i}\left(x\right)\right\}\right)}^{2}}$$

where *m* represents the number of objectives and $f_{i}\left(x\right)$ indicates the *i*th objective value of *x*. The SDE method has been utilized in several MOEAs [27,28,46] as it can assess the quality of a candidate solution (search agent) taking into account both diversity and convergence. Thus, CMOSCA employs the SDE-based distance to measure both diversity and convergence of each search agent in the population.

Afterward, two agents a,b are randomly picked up from the elite agent set *E* and compared in pairs (lines 5–12 of Algorithm 2). The one having a higher fitness value is represented as the winner $x_{w}$, and the other is represented as the loser $x_{l}$. Then the winner is adopted to participate in the position update scheme of the agent (line 14 of Algorithm 2). The position update scheme is defined as follows.

(5) $$\left\{\begin{array}{c} x_{i}^{\prime }=x_{i}+r_{1}\;\times \;\sin\left(r_{2}\right)\;\times \;\left|r_{3}x_{w,i}-x_{i}\right|\;\;r_{4}<0.5\\ x_{i}^{\prime }=x_{i}+r_{1}\;\times \;\cos\left(r_{2}\right)\;\times \;\left|r_{3}x_{w,i}-x_{i}\right|\;\;r_{4}\geq 0.5 \end{array}\right.$$

In the proposed position update scheme, the winner $x_{w}$ is used to replace the best agent (destination point) in the original position update scheme Equation (2) as the best agent does not exist in the MOPs. On the other hand, the winner is close to the Pareto front and has low density since the winner in the elite agent set is chosen by the approach introduced in the NSGA-II. Therefore, the proposed position update scheme with the winner to guide the search of evolution has better convergence and diversity. Note that the $r_{3}$ in Equation (5) is set to 1 to maintain the relationship with the Pareto front.

Finally, the generated new position $x_{i}^{\prime }$ is put into the descendant population $P^{\prime }$. After creating *N* new positions, the process will go back to Algorithm 1 to execute the MOEA environmental selection.

### 3.3. Two Variants of the Proposed CMOSCA

There are two other approaches that can be adopted to measure the quality of candidate agents (solutions) [26,35], i.e., the angle between two agents (solutions) and the Euclidean distance between the origin and the agent. According to these two approaches, we introduce two variants of the proposed CMOSCA, which are denoted as the CMOSCAA and the CMOSCAD, respectively. Algorithms 3 and 4 provide the position update pseudo codes of these two variants. Generally, the angle approach is utilized to evaluate the diversity of the agents, and the distance approach is employed to evaluate the convergence of the agents. However, the SDE approach can measure both diversity and convergence of the agents. Hence, the proposed CMOSCA outperforms the other two variants and the experimental results will confirm this conclusion.
**Algorithm 3** CompetitionBasedSCAPositionUpdate_Angle(*P*)**Input:** current population *P*, the size of elite agent set β.**Output:** descendant population $P^{\prime }$.1:$P^{\prime }$←Φ; /*Choosing the outstanding agents to form the elite agent set*/;2:*E*← Select β agents from *P* relied on the front index and crowding distance.3:**for** each agent $x_{i}$ in *P* **do**4:    {a,b}← Randomly choose two agents from *E*5:    compute the angle $\phi_{1}$ between *a* and $x_{i}$, $\phi_{2}$ between *b* and $x_{i}$6:    **if** $\phi_{2}$ < $\phi_{1}$ **then**7:         $x_{l}=a;$ //loser in the pairwise competition8:         $x_{w}=b;$ //winner in the pairwise competition9:    **else**10:         $x_{l}=b;$11:         $x_{w}=a;$12:    **end if**13:    Update $r_{1}$, $r_{2}$, and $r_{4}$, randomly;14:    Generate new $x_{i}^{\prime }$ by Equation (5);15:    $P^{\prime }$←$P^{\prime }⋃\left\{x_{i}^{\prime }\right\}$;16:**end for**17:**Return**  $P^{\prime }$


**Algorithm 4** CompetitionBasedSCAPositionUpdate_Distance(*P*)**Input:** current population *P*, the size of leader set β.**Output:** descendant population $P^{\prime }$.
1:$P^{\prime }$←Φ; /*Choosing the outstanding agents to form the elite agent set*/;2:*E*← Select β agents from *P* relied on the front index and crowding distance.3:Distance← Calculate the Euclidean distance for each agent in *E*;4:**for** each agent $x_{i}$ in *P* **do**5:    {a,b}← Randomly select two agents from *E*6:    **if** Distance(b) < Distance(a) **then**7:         $x_{l}=a;$ //loser in the pairwise competition8:         $x_{w}=b;$ //winner in the pairwise competition9:    **else**10:         $x_{l}=b;$11:         $x_{w}=a;$12:    **end if**13:    Update $r_{1}$, $r_{2}$, and $r_{4}$, randomly;14:    Generate new $x_{i}^{\prime }$ by Equation (5);15:    $P^{\prime }$←$P^{\prime }⋃\left\{x_{i}^{\prime }\right\}$;16:
**end for**
17:**Return**  $P^{\prime }$


### 3.4. Computational Complexity of the CMOSCA

The time complexity of the CMOSCA is mainly determined by the operations of agent updating strategy and environmental selection. The agent updating strategy mainly composed of creating elite agent set, fitness calculation, and the SCA position update. For a population size *N* and a *m*-objective problem, the elite agents are chosen by non-dominated sorting and crowding distance sorting. The computational complexity of nondominated sorting is $O(m{\left(2N\right)}^{2})$ in the worst case, crowding distance assignment is O(m(2N)log(2N)), and the time complexity of sort on crowded-comparison operator is O(2Nlog(2N)), hence, the overall computational complexity of creating elite agent set is $O\left(m{\left(2N\right)}^{2}+m\left(2N\right)\log\left(2N\right)+2N\log\left(2N\right)\right)∼O\left(mN^{2}\right)$ in the worst case. The time complexity of calculating the fitness of each agent in elite agent set is $O(\beta N^{2})$, where β is the size of elite agent set. The time complexity of the SCA position update is O(N). So the total computational complexity of the agent updating strategy is $O\left(mN^{2}+\beta N^{2}+N\right)∼O\left(mN^{2}\right)$. The time complexity of environmental selection is O(2mNlog(2N)) in the worst case [62]. In summary, the overall computational complexity of one generation in the CMOSCA is $O(mN^{2})$ in the worst case. In the experimental section, we will compare the average runtimes of CMOSCA with that of other six competing MOEAs for MOPs.

## 4. Experimental Studies

In this part, to investigate the effectiveness of our proposed algorithm CMOSCA, we compare it with six typical MOEAs, including EMOSO [28], CMOPSO [26], MOEA/D [29], NSGA-II [20], MOEA/D-DE [32], MMOPSO [64]. These competing methods are chosen because they have shown good performance on MOPs with various types of PF. Where the EMOSO and the CMOPSO are recently proposed methods that also use the competitive mechanism, the MOEA/D and the NSGA-II are two famous algorithms with the GA operators, the MOEA/D-DE is a variant of the MOEA/D with DE operators, and the MMOPSO is an improved version of multi-objective PSO using multiple search strategies. These six MOEAs are programmed with Matlab and embedded into the PlatEMO [65].

We choose 20 test problems, including DTLZ1-DTLZ7 [66], WFG1-WFG9 [67], and ZDT1-ZDT4 [68]. These test problems were designed taking into account diversity characteristics, covering a good representation of various real-world scenarios, such as being disconnected, convex, concave, degenerate, multimodal, and with an irregular Pareto front shape, which can evaluate the efficiency and reliability of the MOEAs. On the other hand, these test problems were widely adopted to validate the performance of the other MOEAs [26,27,28]. Detailed information about these test problems is listed in Table 1. Problems DTLZ and WFG are variable objective functions, and problems ZDT are bi-objective functions. The number of decision variables for problems DTLZ are set to n=k+m−1, where *m* and *n* are the number of objectives and decision variables, respectively. According to the paper [69], *k* is set to 5 for DTLZ1, to 10 for DTLZ2-DTLZ6 and to 20 for DTLZ7. For problems WFG, the number of decision variables is set to n=k+l as recommended in the paper [70], where *k* and *l* are set to m−1 and 10. For problems ZDT, the number of decision variables *n* is set to 30 for ZDT1-ZDT3 and 10 for ZDT4 as suggested in the paper [26].

Inverted generational distance (IGD) [71] and hypervolume (HV) [72] are employed as performance metrics to compare the CMOSCA with other competing MOEAs. To calculate the IGD values, approximately 10,000 reference points are chosen on the real Pareto front of each test problem. Smaller IGD values are better. To calculate the values of the HV, reference points are set to (1,1,…,1) and all objective values are normalized as suggested in the paper [65]. Higher HV values are better.

For the sake of fair comparison, all parameters of the competing methods are set to the values suggested by the original papers. The related parameters employed in the experiments for each method are listed in Table 2. The parameter β of the proposed CMOSCA is set to 5 and it will be analysed in the following section. The population size is set to N=100 for all competing MOEAs. All competing MOEAs utilize maximum number of function evaluations (FES) as the termination criteria. The maximum FES is set to 30,000 for all two- and three-objective problems. All competing MOEAs are independently run 30 times on each test instance, and the mean and the standard deviation of the performance metrics values are reported. In addition, for a comprehensive evaluation, Wilcoxon Signed Rank Test at a significance level of 0.05 was further utilized to test the performance of all competing MOEAs, where the symbols “+”, “=” and “-” indicate that the results of competing MOEAs are statistically superior, similar, and inferior to results obtained by CMOSCA, respectively. All the experiments are performed on a computer with Intel Core i5 @ 3.3 GHz dual-core CPU and Windows 7 operating system with MATLAB 2020b.

### 4.1. Comparisons CMOSCA with Other Competing MOEAs

Table 3 gives the IGD results of the seven competing MOEAs on DTLZ1-DTLZ7, WFG1-WFG9, and ZDT1-ZDT4 test problems. It is obvious that the proposed CMOSCA outperforms the other six competing MOEAs. Specifically, the proposed CMOSCA obtains the best mean IGD results on 15 out of the 36 test problems, the EMOSO on 2 test problems, the CMOPSO on 5 test problems, the MOEA/D on 4 test problems, the NSGA-II on 6 test problems, the MOEA/D-DE on 2 test problems, and the NSGA-II on 2 test problems. The proposed CMOSCA attains better (similar) performances in comparison with the EMOSO on 25 (5) out of the 36 problems. Both CMOSCA and EMOSO adopt the outstanding solutions to guide the search, but they utilize different evolutionary operators. The proposed CMOSCA has better (similar) performances in comparison with the CMOPSO on 19 (6) out of the 36 problems. These two MOEAs have equivalent frameworks, but they employ different competition mechanisms and evolutionary operators, so they perform differently. The proposed CMOSCA has better performances in comparison with the MOEA/D, NSGA-II, MOEA/D-DE, and MMOPSO on 19, 20, 20, and 20 out of the 36 problems, respectively. The CMOSCA obtains better performance mainly owing to the fact that it employs the SDE-based competition mechanism and the SCA evolution operators.

HV results of the seven competing MOEAs are recorded in Table 4. From Table 4, we can clearly observe that our proposed CMOSCA obtains better HV results than the other six competing MOEAs on most of test problems. CMOSCA obtains the best mean HV results on 13 out of 36 problems. The EMOSO, CMOPSO, MOEA/D, NSGA-II, MOEA/D-DE, and MMOPSO obtain the best mean HV results on 5, 1, 5, 7, 2, and 3 out of 36 problems, respectively. It can also be observed from Table 4 that several HV results are zero, which represents that the corresponding MOEA cannot obtain any candidate solution to dominate the reference point on corresponding test problems. For example, our proposed CMOSCA obtains zero HV results on two- and three-objective DTLZ3. This means that the CMOSCA is unable to effectively solve the highly multi-model DTLZ3. EMOSO gets zero HV results on two- and three-objective DTLZ1, two- and three-objective DTLZ3. CMOPSO obtains zero HV results on three-objective DTLZ1, two- and three-objective DTLZ3. The HV result of MMOPSO is zero on two-objective DTLZ3. These experimental results also reflect the limitations of the EMOSO, CMOPSO and MMOPSO.

**Figure 4:**
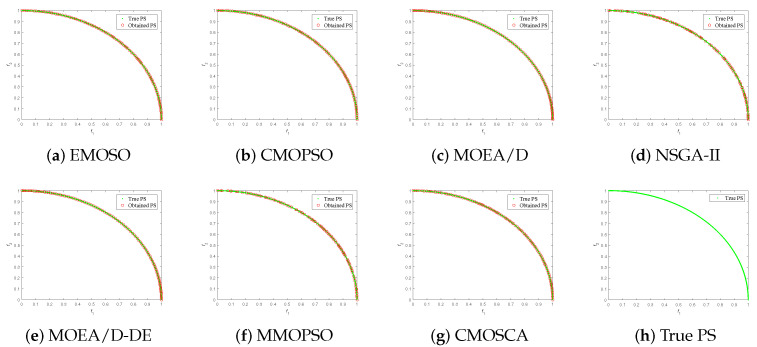
Nondominated solutions obtained by the six competing MOEAs and CMOSCA on two-objective DTLZ6 problem.

**Figure 5:**
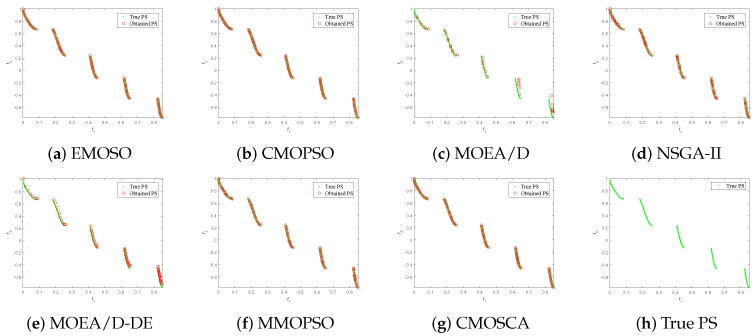
Nondominated solutions obtained by the six competing MOEAs and CMOSCA on two-objective ZDT3 problem.

**Figure 6:**
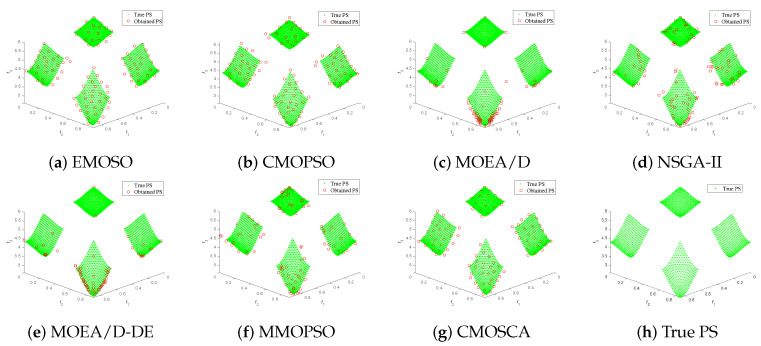
Nondominated solutions obtained by the six competing MOEAs and CMOSCA on three-objective DTLZ7 problem.

**Figure 7:**
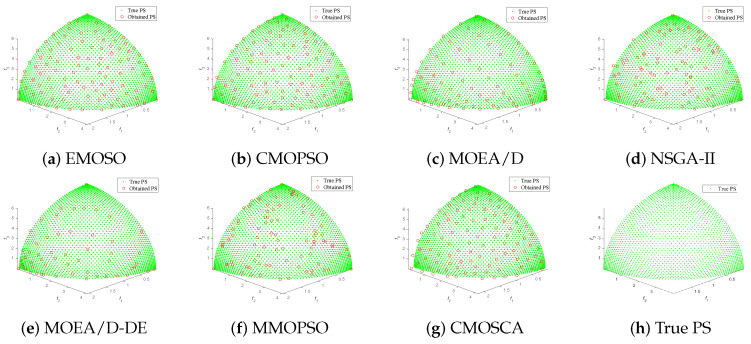
Nondominated solutions obtained by the six competing MOEAs and CMOSCA on three-objective WFG6 problem.

Figure 4, Figure 5, Figure 6 and Figure 7 plot the non-dominated solutions obtained by the CMOSCA and other six competing MOEAs on two-objective DTLZ6 and ZDT3, and three-objective DTLZ7 and WFG6. It can be observed from Figure 4 and Figure 5 that the non-dominated solutions obtained by our proposed CMOSCA on two-objective DTLZ6 and ZDT3 problems have a good distribution and approximate the PF well. This exhibits that the CMOSCA can attain a good trade-off between the diversity and convergence of non-dominated solutions. We can also observe from Figure 6 and Figure 7 that the CMOSCA is able to obtain non-dominated solutions with good diversity and convergence on three-objective problems DTLZ7 and WFG6.

**Figure 8:**
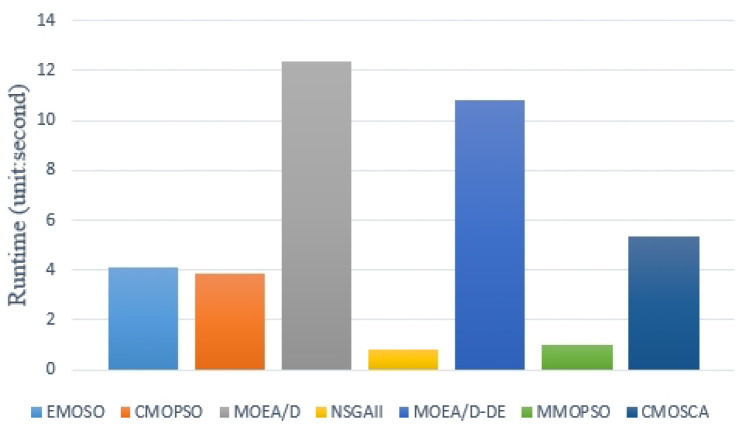
Average runtimes of the all competing methods tested on the all the benchmark problems.

To investigate the computation cost of the CMOSCA, we record the actual running time of those competing MOEAs. Figure 8 shows the average runtimes of the seven competing MOEAs tested on ZDT, DTLZ and WFG series test problems. From Figure 8, we can find that the NSGA-II obtains the best performance in terms of computation cost because the simple dominance relationship selection strategy has significant advantages in real-time computation. We can also find that CMOSCA performs better than MOEA/D and MOEA/D-DE, but weaker than EMOSO, CMOPSO, NSGA-II, and MMOPSO. Hence, we can conclude that the computation time of our proposed CMOSCA is not significantly reduced compared to other competing MOEAs, which is caused by evaluating the SDE fitness values in CMOSCA. However, for MOPs, better performance of the algorithm is more important than computational cost. Therefore, we believe that the computational cost of CMOSCA is acceptable since it can provide decision-makers with a better basis for decision-making.

### 4.2. Parameter Analysis

In the CMOSCA, the parameter β defines the size of elite agent set *E*, which has a great influence on the performance of our proposed CMOSCA, and the outstanding agents in the elite set are adopted to lead the search of the population. Experiments are performed to analyze CMOSCA with different β values (varying from 2 to 30). The other parameter value settings of the CMOSCA are the same as in the previous section. Table 5 reports the experiment IGD values of 30 independent running on DTLZ1-DTLZ7, WFG1-WFG9, and ZDT1-ZDT4 test problems. We can observe from the Table 5 that the CMOSCA using the parameter β=5 has relatively superior results. Table 6 lists the experiment HV values of 30 independent running on DTLZ1-DTLZ7, WFG1-WFG9, and ZDT1-ZDT4 test problems. It can be seen from the Table 6 that CMOSCA using the parameter β=2 has relatively superior results. From these two tables, we can find that the CMOSCA with small β values is better. In this study, we set β to 5 for our proposed CMOSCA to deal with MOPs.

### 4.3. Comparisons among Three CMOSCA Variants

To verify the effects of the SDE-based competition mechanism on the performance of the CMOSCA, the SDE-based competition mechanism is compared with the other two approaches (i.e., angle-based competition mechanism and Euclidean distance-based competition mechanism). The CMOSCA with these two approaches are denoted as the CMOSCAA and the CMOSCAD, respectively. Experiments are performed to compare these three CMOSCA variants. The parameter value settings for these three CMOSCA variants are the same as in the previous section. Table 7 reports the experiment IGD values of 30 independent running on DTLZ1-DTLZ7, WFG1-WFG9 and ZDT1-ZDT4 test problems. It can be observed from the Table 7 that the proposed CMOSCA outperforms the other two variants. Table 8 provides the experiment HV values of 30 independent running on DTLZ1-DTLZ7, WFG1-WFG9 and ZDT1-ZDT4 test problems. From the Table 8, we can also observe that the proposed CMOSCA outperforms the other two variants. This is because the SDE-based competition mechanism can measure both diversity and convergence of the solutions.

### 4.4. Applying Our Proposed CMOSCA to Engineering Design Problems

In this part, to further evaluate the performance of the CMOSCA in handling the real-world problems, we apply the CMOSCA to deal with the engineering design problems including four bar truss design [73], hatch cover design [74], two bar truss design [75], welded beam design [76] and vehicle crashworthiness design [77]. Four bar truss design attempts to minimize the two objective functions (i.e., structural volume and joint displacement) and is subject to the member stresses on the four-bar truss. Hatch cover design is considered as a two-objective problem to minimize the weight of the cover and the constraint violation values [78]. Two bar truss design aims to minimize the two objective functions (i.e., structural volume and joint displacement) and is subject to the member stresses and design variables. Welded beam design is considered as a three-objective problem to minimize the cost, end deflection and the constraint violation values. Vehicle crashworthiness design aims to minimize the three objective functions(i.e., the mass of the vehicle, the integration of collision acceleration and the toe board intrusion). More information on the engineering design problems can be found in the references mentioned above. The proposed CMOSCA and the other six MOEAs are compared on these engineering design problems.

Table 9 provides the statistical HV results obtained by the seven competing MOEAs on these five engineering design problems. From the Table 9, it can be seen that the proposed CMOSCA, MOEA/D-DE and EMOSO obtain the highest HV results on 2, 2, 1 out of 5 engineering design problems, respectively. The other four MOEAs do not have the highest HV results. The proposed CMOSCA performs best on problems four bar truss design and vehicle crashworthiness design. The EMOSO performs best on the hatch cover design problem. The MOEA/D-DE performs best on problems two bar truss design and welded beam design. From the Wilcoxon rank sum test results in the Table 9, it can also be observed that the proposed CMOSCA outperforms the other six MOEAs. Specifically, we can see that CMOSCA outperforms the EMOSO, CMOPSO, MOEA/D, NSGA-II, MOEA/D-DE, and MMOPSO on 2, 2, 3, 4, 3, and 4 out of 5 test problems, respectively, while it loses on 0, 0, 1, 1, 1, and 1 out of 5 test problems. In conclusion, our proposed CMOSCA is a competitive MOEA for solving these engineering design problems.

However, the following aspects need to be considered when using the proposed COMSCA to solve other real-world problems. First, we need to clarify the objectives of the problem and determine the decision variables and constraints involved. Then make appropriate adjustments and improvements to the proposed COMSCA according to the specific conditions of the problem, such as the individual coding method of the algorithm, to meet the needs of the real-world problems.

## 5. Conclusions

A novel multi-objective sine cosine algorithm based on a competitive mechanism (CMOSCA) was presented in this study. In the CMOSCA, a SDE-based competitive mechanism is utilized to devise a new position updating operation for creating offspring agents, which can make use of the SDE to achieve a good balance between the diversity and convergence. Moreover, we also proposed two CMOSCA variants, which adopt the angle-based competitive mechanism and Euclidean distance-based competitive mechanism, respectively. Six representative MOEAs were adopted to verify the performance of the proposed CMOSCA on twenty test problems with different characteristics. We experimentally analyzed the influences of the parameter β and compare the CMOSCA with its two variants. The experimental results exhibited the effectiveness and robustness of our proposed CMOSCA. Finally, several engineering design problems were employed to further evaluate the CMOSCA and results of the engineering problems also demonstrated the competitiveness of our proposed CMOSCA.

However, our proposed CMOSCA algorithm has some shortcomings. First, it does not perform well on some problems with multi-modal PF, such as DTLZ3, because it is still possible to fall into a local optimum. Second, the time complexity of the proposed CMOSCA is not the best among the competing MOEAs. Third, the performance of the proposed CMOSCA may degrade as the number of objectives and decision variables in multi-objective problems increases.

There are many more complex and challenging problems in multi-objective problems, such as variables that are stochastic, uncertain, dynamic, large-scale and constrained, which are interesting and challenging research problems. In our future work, we will further extend our proposed CMOSCA with new strategies [79,80] to solve more complex MOPs, large-scale MOPs, and dynamic MOPs. In addition, we will try to apply our improved algorithms to solve more other real-world problems.

## Figures and Tables

**Table 1:** Characteristics of test problems.

Test Problems	Objective Numbers	Properties of PF
DTLZ1	2,3	Linear
DTLZ2	2,3	Concave, Uni-modal
DTLZ3	2,3	Multimodal
DTLZ4	2,3	Concave, Biased, Uni-modal
DTLZ5	2,3	Concave, Degenerated
DTLZ6	2,3	Concave, Degenerated, Biased
DTLZ7	2,3	Mixed, Disconnected, Scaled
WFG1	2,3	Sharp tails
WFG2	2,3	Disconnected
WFG3	2,3	Mostly degenerated
WFG4-9	2,3	Concave
ZDT1	2	Convex
ZDT2	2	Concave
ZDT3	2	Disconnected, Multimodal
ZDT4	2	Convex, Multimodal

**Table 2:** Parameters Settings of all competing MOEAs.

Method	Parameters Settings
EMOSO	$NL=4,c_{1},c_{2}\in \left[0,1\right],r_{1},r_{2},r_{3},r_{4},r_{5},r_{6}\in \left[0,1\right],p_{m}=1/D,\eta_{m}=20,\gamma =5$
CMOPSO	$\omega ,r_{1}\in \left[0,1\right],p_{m}=1/D,\eta_{m}=20,\gamma =10$
MOEA/D	$p_{c}=1,p_{m}=1/D,\eta_{c}=\eta_{m}=20,T=0.1*N$
NSGA-II	$p_{c}=1,p_{m}=1/D,\eta_{c}=\eta_{m}=20$
MOEA/D-DE	$CR=1,F=0.5,\delta =0.9,nr=2,\eta_{m}=20,T=0.1*N$
MMOPSO	$\omega \in \left[0.1,0.5\right],c_{1},c_{2}\in \left[1.5,2.5\right],r_{1},r_{2}\in \left[0,1\right],$
	$p_{c}=0.9,p_{m}=1/D,\eta_{c}=\eta_{m}=20$
CMOSCA	$r_{1}\in \left[0,2\right],r_{2}\in \left[0,2\pi \right],r_{3}=1,r_{4}\in \left[0,1\right],\beta =5$

**Table 3:** IGD values achieved by CMOSCA and six competing MOEAs.

Problem	M	D	EMOSO	CMOPSO	MOEAD	CMOSCA
DTLZ1	2	6	1.7772 $\;\times \;10^{1}$ (2.97$\;\times \;10^{0}$) =	8.0014$\;\times \;10^{-1}$ (1.15$\;\times \;10^{0}$) +	2.6325$\;\times \;10^{-3}$ (7.36$\;\times \;10^{-4}$) +	1.8233 $\;\times \;10^{1}$ (6.02$\;\times \;10^{0}$)
	3	7	1.6639 $\;\times \;10^{1}$ (2.89$\;\times \;10^{0}$) -	8.2647$\;\times \;10^{0}$ (4.52$\;\times \;10^{0}$) +	**2.1353$\;\times \;10^{-2}$ (1.59$\;\times \;10^{-3}$) +**	1.1081 $\;\times \;10^{1}$ (4.81$\;\times \;10^{0}$)
DTLZ2	2	11	4.3749$\;\times \;10^{-3}$ (6.68$\;\times \;10^{-5}$) -	4.3891$\;\times \;10^{-3}$ (6.81$\;\times \;10^{-5}$) -	**3.9697$\;\times \;10^{-3}$ (1.32$\;\times \;10^{-5}$) +**	4.1149$\;\times \;10^{-3}$ (3.78$\;\times \;10^{-5}$)
	3	12	5.8551$\;\times \;10^{-2}$ (9.79$\;\times \;10^{-4}$) -	5.7573$\;\times \;10^{-2}$ (9.46$\;\times \;10^{-4}$) -	5.4467$\;\times \;10^{-2}$ (1.71$\;\times \;10^{-6}$) -	**5.3243$\;\times \;10^{-2}$ (4.56$\;\times \;10^{-4}$)**
DTLZ3	2	11	1.8287$\;\times \;10^{2}$ (1.26 $\;\times \;10^{1}$) -	3.2331 $\;\times \;10^{1}$ (1.91 $\;\times \;10^{1}$) +	9.9329$\;\times \;10^{-2}$ (2.00$\;\times \;10^{-1}$) +	1.5902$\;\times \;10^{2}$ (1.94 $\;\times \;10^{1}$)
	3	12	1.8301$\;\times \;10^{2}$ (1.44 $\;\times \;10^{1}$) -	8.9128 $\;\times \;10^{1}$ (4.24 $\;\times \;10^{1}$) +	7.5061$\;\times \;10^{-1}$ (8.10$\;\times \;10^{-1}$) +	1.6203$\;\times \;10^{2}$ (1.60 $\;\times \;10^{1}$)
DTLZ4	2	11	1.7657$\;\times \;10^{-1}$ (3.17$\;\times \;10^{-1}$) -	1.7657$\;\times \;10^{-1}$ (3.17$\;\times \;10^{-1}$) -	2.5001$\;\times \;10^{-1}$ (3.54$\;\times \;10^{-1}$) -	**4.1119$\;\times \;10^{-3}$ (3.26$\;\times \;10^{-5}$)**
	3	12	9.8710$\;\times \;10^{-2}$ (9.90$\;\times \;10^{-3}$) -	1.2015$\;\times \;10^{-1}$ (2.24$\;\times \;10^{-1}$) -	3.9264$\;\times \;10^{-1}$ (3.35$\;\times \;10^{-1}$) =	**5.9577$\;\times \;10^{-2}$ (1.47$\;\times \;10^{-3}$)**
DTLZ5	2	11	4.4092$\;\times \;10^{-3}$ (6.13$\;\times \;10^{-5}$) -	4.3778$\;\times \;10^{-3}$ (5.31$\;\times \;10^{-5}$) -	**3.9714$\;\times \;10^{-3}$ (1.59$\;\times \;10^{-5}$) +**	4.1107$\;\times \;10^{-3}$ (2.65$\;\times \;10^{-5}$)
	3	12	7.6799$\;\times \;10^{-3}$ (9.41$\;\times \;10^{-4}$) -	6.5749$\;\times \;10^{-3}$ (6.23$\;\times \;10^{-4}$) -	3.3783$\;\times \;10^{-2}$ (5.81$\;\times \;10^{-5}$) -	**4.3665$\;\times \;10^{-3}$ (1.69$\;\times \;10^{-4}$)**
DTLZ6	2	11	4.1288$\;\times \;10^{-3}$ (3.15$\;\times \;10^{-5}$) =	4.1243$\;\times \;10^{-3}$ (3.07$\;\times \;10^{-5}$) =	**3.9659$\;\times \;10^{-3}$ (7.10$\;\times \;10^{-7}$) +**	4.1140$\;\times \;10^{-3}$ (3.03$\;\times \;10^{-5}$)
	3	12	4.1934$\;\times \;10^{-3}$ (4.70$\;\times \;10^{-5}$) =	4.2017$\;\times \;10^{-3}$ (5.73$\;\times \;10^{-5}$) =	3.3854$\;\times \;10^{-2}$ (9.78$\;\times \;10^{-5}$) -	**4.1788$\;\times \;10^{-3}$ (5.83$\;\times \;10^{-5}$)**
DTLZ7	2	21	3.3686$\;\times \;10^{-2}$ (1.11$\;\times \;10^{-1}$) -	3.3711$\;\times \;10^{-2}$ (1.11$\;\times \;10^{-1}$) =	1.2727$\;\times \;10^{-1}$ (1.96$\;\times \;10^{-1}$) -	**4.5057$\;\times \;10^{-3}$ (5.13$\;\times \;10^{-5}$)**
	3	22	6.4654$\;\times \;10^{-2}$ (1.67$\;\times \;10^{-3}$) -	1.3352$\;\times \;10^{-1}$ (1.95$\;\times \;10^{-1}$) -	1.9754$\;\times \;10^{-1}$ (1.64$\;\times \;10^{-1}$) -	**5.8941$\;\times \;10^{-2}$ (6.68$\;\times \;10^{-4}$)**
WFG1	2	11	1.4596$\;\times \;10^{0}$ (3.48$\;\times \;10^{-2}$) -	6.6972$\;\times \;10^{-1}$ (1.00$\;\times \;10^{-1}$) -	1.6275$\;\times \;10^{-1}$ (2.19$\;\times \;10^{-2}$) +	3.8033$\;\times \;10^{-1}$ (1.19$\;\times \;10^{-1}$)
	3	12	1.8478$\;\times \;10^{0}$ (4.27$\;\times \;10^{-2}$) -	1.4486$\;\times \;10^{0}$ (5.24$\;\times \;10^{-2}$) -	3.2387$\;\times \;10^{-1}$ (3.54$\;\times \;10^{-2}$) +	8.5394$\;\times \;10^{-1}$ (1.43$\;\times \;10^{-1}$)
WFG2	2	11	1.2332$\;\times \;10^{-2}$ (4.29$\;\times \;10^{-4}$) =	**1.1911$\;\times \;10^{-2}$ (3.63$\;\times \;10^{-4}$) =**	6.9447$\;\times \;10^{-2}$ (4.10$\;\times \;10^{-2}$) -	3.5635$\;\times \;10^{-2}$ (5.38$\;\times \;10^{-2}$)
	3	12	1.8572$\;\times \;10^{-1}$ (6.77$\;\times \;10^{-3}$) +	**1.7966$\;\times \;10^{-1}$ (4.66$\;\times \;10^{-3}$) =**	2.5232$\;\times \;10^{-1}$ (1.07$\;\times \;10^{-2}$) -	1.9064$\;\times \;10^{-1}$ (3.08$\;\times \;10^{-2}$)
WFG3	2	11	**1.3828$\;\times \;10^{-2}$ (3.72$\;\times \;10^{-4}$) +**	1.3974$\;\times \;10^{-2}$ (3.74$\;\times \;10^{-4}$) +	2.2398$\;\times \;10^{-2}$ (3.39$\;\times \;10^{-3}$) +	2.9280$\;\times \;10^{-2}$ (5.16$\;\times \;10^{-2}$)
	3	12	2.0509$\;\times \;10^{-1}$ (1.10$\;\times \;10^{-2}$) -	1.5467$\;\times \;10^{-1}$ (1.40$\;\times \;10^{-2}$) -	1.7054$\;\times \;10^{-1}$ (1.83$\;\times \;10^{-2}$) -	1.3883$\;\times \;10^{-1}$ (6.04$\;\times \;10^{-2}$)
WFG4	2	11	6.9249$\;\times \;10^{-2}$ (4.63$\;\times \;10^{-3}$) -	4.5178$\;\times \;10^{-2}$ (1.29$\;\times \;10^{-2}$) -	3.1383$\;\times \;10^{-2}$ (4.21$\;\times \;10^{-3}$) -	2.0532$\;\times \;10^{-2}$ (4.51$\;\times \;10^{-3}$)
	3	12	2.6565$\;\times \;10^{-1}$ (5.11$\;\times \;10^{-3}$) -	2.6055$\;\times \;10^{-1}$ (5.32$\;\times \;10^{-3}$) -	2.6299$\;\times \;10^{-1}$ (6.02$\;\times \;10^{-3}$) -	**2.4095$\;\times \;10^{-1}$ (8.39$\;\times \;10^{-3}$)**
WFG5	2	11	6.6847$\;\times \;10^{-2}$ (1.72$\;\times \;10^{-3}$) -	6.7869$\;\times \;10^{-2}$ (2.04$\;\times \;10^{-3}$) -	7.1356$\;\times \;10^{-2}$ (1.40$\;\times \;10^{-3}$) -	**6.3608$\;\times \;10^{-2}$ (1.83$\;\times \;10^{-4}$)**
	3	12	2.4004$\;\times \;10^{-1}$ (4.84$\;\times \;10^{-3}$) -	2.4796$\;\times \;10^{-1}$ (6.45$\;\times \;10^{-3}$) -	2.5268$\;\times \;10^{-1}$ (3.45$\;\times \;10^{-3}$) -	**2.2262$\;\times \;10^{-1}$ (3.68$\;\times \;10^{-3}$)**
WFG6	2	11	1.9095$\;\times \;10^{-2}$ (8.98$\;\times \;10^{-3}$) +	**1.8875$\;\times \;10^{-2}$ (5.49$\;\times \;10^{-3}$) +**	9.9941$\;\times \;10^{-2}$ (2.20$\;\times \;10^{-2}$) +	2.2547$\;\times \;10^{-1}$ (1.68$\;\times \;10^{-5}$)
	3	12	**2.3183$\;\times \;10^{-1}$ (5.23$\;\times \;10^{-3}$) +**	2.4133$\;\times \;10^{-1}$ (7.05$\;\times \;10^{-3}$) +	2.9133$\;\times \;10^{-1}$ (1.67$\;\times \;10^{-2}$) +	3.3555$\;\times \;10^{-1}$ (1.77$\;\times \;10^{-3}$)
WFG7	2	11	1.4701$\;\times \;10^{-2}$ (3.70$\;\times \;10^{-4}$) -	1.4495$\;\times \;10^{-2}$ (3.87$\;\times \;10^{-4}$) -	2.9914$\;\times \;10^{-2}$ (3.85$\;\times \;10^{-3}$) -	**1.2639$\;\times \;10^{-2}$ (1.05$\;\times \;10^{-4}$)**
	3	12	2.2766$\;\times \;10^{-1}$ (3.31$\;\times \;10^{-3}$) -	2.3370$\;\times \;10^{-1}$ (4.57$\;\times \;10^{-3}$) -	3.4056$\;\times \;10^{-1}$ (4.60$\;\times \;10^{-2}$) -	**2.1066$\;\times \;10^{-1}$ (1.88$\;\times \;10^{-3}$)**
WFG8	2	11	1.2258$\;\times \;10^{-1}$ (3.57$\;\times \;10^{-3}$) -	1.1731$\;\times \;10^{-1}$ (3.10$\;\times \;10^{-3}$) =	1.2890$\;\times \;10^{-1}$ (1.08$\;\times \;10^{-2}$) -	1.1660$\;\times \;10^{-1}$ (3.17$\;\times \;10^{-3}$)
	3	12	3.4539$\;\times \;10^{-1}$ (7.05$\;\times \;10^{-3}$) -	3.3864$\;\times \;10^{-1}$ (7.18$\;\times \;10^{-3}$) -	3.2357$\;\times \;10^{-1}$ (1.07$\;\times \;10^{-2}$) -	**3.1606$\;\times \;10^{-1}$ (9.77$\;\times \;10^{-3}$)**
WFG9	2	11	2.8963$\;\times \;10^{-2}$ (2.07$\;\times \;10^{-3}$) +	2.6831$\;\times \;10^{-2}$ (1.65$\;\times \;10^{-3}$) +	7.5073$\;\times \;10^{-2}$ (6.03$\;\times \;10^{-2}$) +	2.2676$\;\times \;10^{-1}$ (2.47$\;\times \;10^{-3}$)
	3	12	2.3940$\;\times \;10^{-1}$ (4.84$\;\times \;10^{-2}$) +	**2.2124$\;\times \;10^{-1}$ (4.13$\;\times \;10^{-3}$) +**	2.9889$\;\times \;10^{-1}$ (3.10$\;\times \;10^{-2}$) +	3.4464$\;\times \;10^{-1}$ (3.88$\;\times \;10^{-3}$)
ZDT1	2	30	4.0907$\;\times \;10^{-3}$ (6.75$\;\times \;10^{-5}$) -	4.1992$\;\times \;10^{-3}$ (1.03$\;\times \;10^{-4}$) -	1.3219$\;\times \;10^{-2}$ (1.17$\;\times \;10^{-2}$) -	**3.8994$\;\times \;10^{-3}$ (4.98$\;\times \;10^{-5}$)**
ZDT2	2	30	4.0207$\;\times \;10^{-3}$ (6.83$\;\times \;10^{-5}$) -	4.1095$\;\times \;10^{-3}$ (7.72$\;\times \;10^{-5}$) -	2.9084$\;\times \;10^{-2}$ (3.89$\;\times \;10^{-2}$) -	**3.8933$\;\times \;10^{-3}$ (3.48$\;\times \;10^{-5}$)**
ZDT3	2	30	4.6658$\;\times \;10^{-3}$ (8.52$\;\times \;10^{-5}$) =	**4.6397$\;\times \;10^{-3}$ (6.51$\;\times \;10^{-5}$) +**	3.1477$\;\times \;10^{-2}$ (2.11$\;\times \;10^{-2}$) -	4.6982$\;\times \;10^{-3}$ (7.19$\;\times \;10^{-5}$)
ZDT4	2	10	1.7673 $\;\times \;10^{1}$ (4.62$\;\times \;10^{0}$) -	3.0164$\;\times \;10^{-1}$ (3.89$\;\times \;10^{-1}$) +	2.3644$\;\times \;10^{-2}$ (1.66$\;\times \;10^{-2}$) +	1.7501 $\;\times \;10^{1}$ (1.63 $\;\times \;10^{1}$)
+/-/=			6/25/5	11/19/6	15/20/1	
**Problem**	**M**	**D**	**NSGAII**	**MOEADDE**	**MMOPSO**	**CMOSCA**
DTLZ1	2	6	2.4598$\;\times \;10^{-3}$ (3.99$\;\times \;10^{-4}$) +	**2.3927$\;\times \;10^{-3}$ (1.30$\;\times \;10^{-3}$) +**	9.1228$\;\times \;10^{-3}$ (3.15$\;\times \;10^{-2}$) +	1.8233 $\;\times \;10^{1}$ (6.02$\;\times \;10^{0}$)
	3	7	3.7968$\;\times \;10^{-2}$ (4.95$\;\times \;10^{-2}$) +	7.2322$\;\times \;10^{-2}$ (9.75$\;\times \;10^{-2}$) +	3.5135$\;\times \;10^{-1}$ (2.76$\;\times \;10^{-1}$) +	1.1081 $\;\times \;10^{1}$ (4.81$\;\times \;10^{0}$)
DTLZ2	2	11	5.0930$\;\times \;10^{-3}$ (1.71$\;\times \;10^{-4}$) -	3.9778$\;\times \;10^{-3}$ (5.04$\;\times \;10^{-6}$) +	5.3158$\;\times \;10^{-3}$ (5.06$\;\times \;10^{-4}$) -	4.1149$\;\times \;10^{-3}$ (3.78$\;\times \;10^{-5}$)
	3	12	7.2640$\;\times \;10^{-2}$ (2.81$\;\times \;10^{-3}$) -	7.6176$\;\times \;10^{-2}$ (1.11$\;\times \;10^{-3}$) -	7.1806$\;\times \;10^{-2}$ (3.05$\;\times \;10^{-3}$) -	**5.3243$\;\times \;10^{-2}$ (4.56$\;\times \;10^{-4}$)**
DTLZ3	2	11	**6.4469$\;\times \;10^{-2}$ (1.99$\;\times \;10^{-1}$) +**	1.0659 $\;\times \;10^{1}$ (1.22 $\;\times \;10^{1}$) +	9.3097$\;\times \;10^{0}$ (7.97$\;\times \;10^{0}$) +	1.5902$\;\times \;10^{2}$ (1.94 $\;\times \;10^{1}$)
	3	12	**2.3057$\;\times \;10^{-1}$ (3.63$\;\times \;10^{-1}$) +**	4.5983$\;\times \;10^{0}$ (7.79$\;\times \;10^{0}$) +	1.3998 $\;\times \;10^{1}$ (9.70$\;\times \;10^{0}$) +	1.6203$\;\times \;10^{2}$ (1.60 $\;\times \;10^{1}$)
DTLZ4	2	11	1.0339$\;\times \;10^{-1}$ (2.55$\;\times \;10^{-1}$) -	4.1241$\;\times \;10^{-3}$ (9.48$\;\times \;10^{-5}$) =	1.2797$\;\times \;10^{-1}$ (2.79$\;\times \;10^{-1}$) -	**4.1119$\;\times \;10^{-3}$ (3.26$\;\times \;10^{-5}$)**
	3	12	9.9949$\;\times \;10^{-2}$ (1.60$\;\times \;10^{-1}$) -	1.2732$\;\times \;10^{-1}$ (6.97$\;\times \;10^{-2}$) -	7.0944$\;\times \;10^{-2}$ (2.98$\;\times \;10^{-3}$) -	**5.9577$\;\times \;10^{-2}$ (1.47$\;\times \;10^{-3}$)**
DTLZ5	2	11	5.0880$\;\times \;10^{-3}$ (2.22$\;\times \;10^{-4}$) -	3.9799$\;\times \;10^{-3}$ (9.64$\;\times \;10^{-6}$) +	5.2739$\;\times \;10^{-3}$ (2.28$\;\times \;10^{-4}$) -	4.1107$\;\times \;10^{-3}$ (2.65$\;\times \;10^{-5}$)
	3	12	6.3135$\;\times \;10^{-3}$ (3.30$\;\times \;10^{-4}$) -	1.4368$\;\times \;10^{-2}$ (9.98$\;\times \;10^{-5}$) -	6.2533$\;\times \;10^{-3}$ (4.41$\;\times \;10^{-4}$) -	**4.3665$\;\times \;10^{-3}$ (1.69$\;\times \;10^{-4}$)**
DTLZ6	2	11	5.6922$\;\times \;10^{-3}$ (3.31$\;\times \;10^{-4}$) -	3.9664$\;\times \;10^{-3}$ (7.16$\;\times \;10^{-8}$) +	5.6898$\;\times \;10^{-3}$ (4.63$\;\times \;10^{-4}$) -	4.1140$\;\times \;10^{-3}$ (3.03$\;\times \;10^{-5}$)
	3	12	6.4813$\;\times \;10^{-3}$ (3.20$\;\times \;10^{-4}$) -	1.4503$\;\times \;10^{-2}$ (5.27$\;\times \;10^{-5}$) -	6.8147$\;\times \;10^{-3}$ (8.01$\;\times \;10^{-4}$) -	**4.1788$\;\times \;10^{-3}$ (5.83$\;\times \;10^{-5}$)**
DTLZ7	2	21	5.2692$\;\times \;10^{-3}$ (1.42$\;\times \;10^{-4}$) -	9.4450$\;\times \;10^{-2}$ (1.78$\;\times \;10^{-1}$) -	1.8044$\;\times \;10^{-1}$ (2.18$\;\times \;10^{-1}$) -	**4.5057$\;\times \;10^{-3}$ (5.13$\;\times \;10^{-5}$)**
	3	22	7.8673$\;\times \;10^{-2}$ (3.90$\;\times \;10^{-3}$) -	2.5102$\;\times \;10^{-1}$ (1.37$\;\times \;10^{-1}$) -	1.5383$\;\times \;10^{-1}$ (1.57$\;\times \;10^{-1}$) -	**5.8941$\;\times \;10^{-2}$ (6.68$\;\times \;10^{-4}$)**
WFG1	2	11	**8.3736$\;\times \;10^{-2}$ (3.38$\;\times \;10^{-2}$) +**	3.9448$\;\times \;10^{-1}$ (9.68$\;\times \;10^{-2}$) =	1.1203$\;\times \;10^{-1}$ (5.09$\;\times \;10^{-2}$) +	3.8033$\;\times \;10^{-1}$ (1.19$\;\times \;10^{-1}$)
	3	12	**2.3801$\;\times \;10^{-1}$ (1.60$\;\times \;10^{-2}$) +**	1.1987$\;\times \;10^{0}$ (1.32$\;\times \;10^{-1}$) -	3.8707$\;\times \;10^{-1}$ (4.84$\;\times \;10^{-2}$) +	8.5394$\;\times \;10^{-1}$ (1.43$\;\times \;10^{-1}$)
WFG2	2	11	1.2893$\;\times \;10^{-2}$ (5.50$\;\times \;10^{-4}$) +	2.2462$\;\times \;10^{-2}$ (7.29$\;\times \;10^{-4}$) +	1.2785$\;\times \;10^{-2}$ (5.11$\;\times \;10^{-4}$) +	3.5635$\;\times \;10^{-2}$ (5.38$\;\times \;10^{-2}$)
	3	12	2.3473$\;\times \;10^{-1}$ (1.62$\;\times \;10^{-2}$) -	3.4041$\;\times \;10^{-1}$ (1.91$\;\times \;10^{-2}$) -	2.2874$\;\times \;10^{-1}$ (1.07$\;\times \;10^{-2}$) -	1.9064$\;\times \;10^{-1}$ (3.08$\;\times \;10^{-2}$)
WFG3	2	11	1.5434$\;\times \;10^{-2}$ (7.48$\;\times \;10^{-4}$) +	1.5965$\;\times \;10^{-2}$ (7.23$\;\times \;10^{-4}$) +	1.4776$\;\times \;10^{-2}$ (6.86$\;\times \;10^{-4}$) +	2.9280$\;\times \;10^{-2}$ (5.16$\;\times \;10^{-2}$)
	3	12	1.0219$\;\times \;10^{-1}$ (1.42$\;\times \;10^{-2}$) +	1.7134$\;\times \;10^{-1}$ (2.62$\;\times \;10^{-2}$) -	**9.9848$\;\times \;10^{-2}$ (2.37$\;\times \;10^{-2}$) +**	1.3883$\;\times \;10^{-1}$ (6.04$\;\times \;10^{-2}$)
WFG4	2	11	**1.5646$\;\times \;10^{-2}$ (6.48$\;\times \;10^{-4}$) +**	5.1517$\;\times \;10^{-2}$ (8.41$\;\times \;10^{-3}$) -	1.7160$\;\times \;10^{-2}$ (1.10$\;\times \;10^{-3}$) +	2.0532$\;\times \;10^{-2}$ (4.51$\;\times \;10^{-3}$)
	3	12	2.8260$\;\times \;10^{-1}$ (8.38$\;\times \;10^{-3}$) -	3.8857$\;\times \;10^{-1}$ (8.12$\;\times \;10^{-3}$) -	3.0067$\;\times \;10^{-1}$ (1.15$\;\times \;10^{-2}$) -	**2.4095$\;\times \;10^{-1}$ (8.39$\;\times \;10^{-3}$)**
WFG5	2	11	6.5711$\;\times \;10^{-2}$ (1.52$\;\times \;10^{-3}$) -	6.8569$\;\times \;10^{-2}$ (1.90$\;\times \;10^{-3}$) -	6.6765$\;\times \;10^{-2}$ (2.47$\;\times \;10^{-3}$) -	**6.3608$\;\times \;10^{-2}$ (1.83$\;\times \;10^{-4}$)**
	3	12	2.8644$\;\times \;10^{-1}$ (9.22$\;\times \;10^{-3}$) -	3.3799$\;\times \;10^{-1}$ (5.88$\;\times \;10^{-3}$) -	2.8841$\;\times \;10^{-1}$ (1.13$\;\times \;10^{-2}$) -	**2.2262$\;\times \;10^{-1}$ (3.68$\;\times \;10^{-3}$)**
WFG6	2	11	7.7548$\;\times \;10^{-2}$ (1.95$\;\times \;10^{-2}$) +	6.4813$\;\times \;10^{-2}$ (7.48$\;\times \;10^{-2}$) +	6.0904$\;\times \;10^{-2}$ (6.81$\;\times \;10^{-2}$) +	2.2547$\;\times \;10^{-1}$ (1.68$\;\times \;10^{-5}$)
	3	12	3.1640$\;\times \;10^{-1}$ (1.97$\;\times \;10^{-2}$) +	3.9947$\;\times \;10^{-1}$ (3.51$\;\times \;10^{-2}$) -	3.3096$\;\times \;10^{-1}$ (5.51$\;\times \;10^{-2}$) +	3.3555$\;\times \;10^{-1}$ (1.77$\;\times \;10^{-3}$)
WFG7	2	11	1.7271$\;\times \;10^{-2}$ (7.33$\;\times \;10^{-4}$) -	1.4203$\;\times \;10^{-2}$ (3.44$\;\times \;10^{-4}$) -	1.6735$\;\times \;10^{-2}$ (1.51$\;\times \;10^{-3}$) -	**1.2639$\;\times \;10^{-2}$ (1.05$\;\times \;10^{-4}$)**
	3	12	2.9255$\;\times \;10^{-1}$ (1.31$\;\times \;10^{-2}$) -	3.5950$\;\times \;10^{-1}$ (5.15$\;\times \;10^{-3}$) -	2.8503$\;\times \;10^{-1}$ (1.25$\;\times \;10^{-2}$) -	**2.1066$\;\times \;10^{-1}$ (1.88$\;\times \;10^{-3}$)**
WFG8	2	11	1.1129$\;\times \;10^{-1}$ (1.44$\;\times \;10^{-3}$) +	**1.0641$\;\times \;10^{-1}$ (4.83$\;\times \;10^{-3}$) +**	1.1043$\;\times \;10^{-1}$ (2.34$\;\times \;10^{-3}$) +	1.1660$\;\times \;10^{-1}$ (3.17$\;\times \;10^{-3}$)
	3	12	3.7766$\;\times \;10^{-1}$ (1.28$\;\times \;10^{-2}$) -	4.2707$\;\times \;10^{-1}$ (1.17$\;\times \;10^{-2}$) -	3.6982$\;\times \;10^{-1}$ (1.18$\;\times \;10^{-2}$) -	**3.1606$\;\times \;10^{-1}$ (9.77$\;\times \;10^{-3}$)**
WFG9	2	11	2.7899$\;\times \;10^{-2}$ (3.79$\;\times \;10^{-2}$) +	2.8731$\;\times \;10^{-2}$ (2.89$\;\times \;10^{-3}$) +	**2.3062$\;\times \;10^{-2}$ (2.93$\;\times \;10^{-3}$) +**	2.2676$\;\times \;10^{-1}$ (2.47$\;\times \;10^{-3}$)
	3	12	3.0118$\;\times \;10^{-1}$ (3.33$\;\times \;10^{-2}$) +	3.3531$\;\times \;10^{-1}$ (4.42$\;\times \;10^{-3}$) +	2.8868$\;\times \;10^{-1}$ (2.06$\;\times \;10^{-2}$) +	3.4464$\;\times \;10^{-1}$ (3.88$\;\times \;10^{-3}$)
ZDT1	2	30	4.7757$\;\times \;10^{-3}$ (1.65$\;\times \;10^{-4}$) -	1.2678$\;\times \;10^{-2}$ (3.54$\;\times \;10^{-3}$) -	4.8826$\;\times \;10^{-3}$ (2.46$\;\times \;10^{-4}$) -	**3.8994$\;\times \;10^{-3}$ (4.98$\;\times \;10^{-5}$)**
ZDT2	2	30	4.8999$\;\times \;10^{-3}$ (1.74$\;\times \;10^{-4}$) -	8.5343$\;\times \;10^{-3}$ (1.84$\;\times \;10^{-3}$) -	5.1728$\;\times \;10^{-3}$ (2.42$\;\times \;10^{-4}$) -	**3.8933$\;\times \;10^{-3}$ (3.48$\;\times \;10^{-5}$)**
ZDT3	2	30	6.4385$\;\times \;10^{-3}$ (5.32$\;\times \;10^{-3}$) -	1.7481$\;\times \;10^{-2}$ (6.53$\;\times \;10^{-3}$) -	5.5287$\;\times \;10^{-3}$ (2.89$\;\times \;10^{-4}$) -	4.6982$\;\times \;10^{-3}$ (7.19$\;\times \;10^{-5}$)
ZDT4	2	10	**5.3574$\;\times \;10^{-3}$ (7.20$\;\times \;10^{-4}$) +**	1.9761$\;\times \;10^{-1}$ (1.30$\;\times \;10^{-1}$) +	2.1125$\;\times \;10^{-2}$ (4.84$\;\times \;10^{-2}$) +	1.7501 $\;\times \;10^{1}$ (1.63 $\;\times \;10^{1}$)
+/-/=			16/20/0	14/20/2	16/20/0	

“+”, “=” and “-” indicate that the results of competing MOEAs are statistically superior, similar, and inferior to results obtained by the CMOSCA, respectively. The best result of each test problem is displayed in bold.

**Table 4:** HV values achieved by CMOSCA and six competing MOEAs.

Problem	M	D	EMOSO	CMOPSO	MOEAD	CMOSCA
DTLZ1	2	6	0.0000$\;\times \;10^{0}$ (0.00$\;\times \;10^{0}$) =	1.5837$\;\times \;10^{-1}$ (2.39$\;\times \;10^{-1}$) +	5.7877$\;\times \;10^{-1}$ (2.40$\;\times \;10^{-3}$) +	0.0000$\;\times \;10^{0}$ (0.00$\;\times \;10^{0}$)
	3	7	0.0000$\;\times \;10^{0}$ (0.00$\;\times \;10^{0}$) =	0.0000$\;\times \;10^{0}$ (0.00$\;\times \;10^{0}$) =	**8.3698$\;\times \;10^{-1}$ (5.86$\;\times \;10^{-3}$) +**	0.0000$\;\times \;10^{0}$ (0.00$\;\times \;10^{0}$)
DTLZ2	2	11	3.4650$\;\times \;10^{-1}$ (1.66$\;\times \;10^{-4}$) -	3.4644$\;\times \;10^{-1}$ (1.89$\;\times \;10^{-4}$) -	3.4720$\;\times \;10^{-1}$ (5.03$\;\times \;10^{-6}$) -	**3.4743$\;\times \;10^{-1}$ (5.68$\;\times \;10^{-5}$)**
	3	12	5.4144$\;\times \;10^{-1}$ (3.16$\;\times \;10^{-3}$) -	5.4220$\;\times \;10^{-1}$ (2.65$\;\times \;10^{-3}$) -	**5.5947$\;\times \;10^{-1}$ (3.80$\;\times \;10^{-5}$) +**	5.5278$\;\times \;10^{-1}$ (1.94$\;\times \;10^{-3}$)
DTLZ3	2	11	0.0000$\;\times \;10^{0}$ (0.00$\;\times \;10^{0}$) =	0.0000$\;\times \;10^{0}$ (0.00$\;\times \;10^{0}$) =	2.5915$\;\times \;10^{-1}$ (6.32$\;\times \;10^{-2}$) +	0.0000$\;\times \;10^{0}$ (0.00$\;\times \;10^{0}$)
	3	12	0.0000$\;\times \;10^{0}$ (0.00$\;\times \;10^{0}$) =	0.0000$\;\times \;10^{0}$ (0.00$\;\times \;10^{0}$) =	2.0017$\;\times \;10^{-1}$ (2.09$\;\times \;10^{-1}$) +	0.0000$\;\times \;10^{0}$ (0.00$\;\times \;10^{0}$)
DTLZ4	2	11	2.8682$\;\times \;10^{-1}$ (1.10$\;\times \;10^{-1}$) -	2.8669$\;\times \;10^{-1}$ (1.10$\;\times \;10^{-1}$) -	2.6177$\;\times \;10^{-1}$ (1.23$\;\times \;10^{-1}$) -	**3.4733$\;\times \;10^{-1}$ (7.32$\;\times \;10^{-5}$)**
	3	12	5.3362$\;\times \;10^{-1}$ (3.36$\;\times \;10^{-3}$) -	5.0357$\;\times \;10^{-1}$ (1.12$\;\times \;10^{-1}$) -	3.9433$\;\times \;10^{-1}$ (1.71$\;\times \;10^{-1}$) =	**5.3966$\;\times \;10^{-1}$ (3.31$\;\times \;10^{-3}$)**
DTLZ5	2	11	3.4643$\;\times \;10^{-1}$ (1.26$\;\times \;10^{-4}$) -	3.4643$\;\times \;10^{-1}$ (1.22$\;\times \;10^{-4}$) -	3.4720$\;\times \;10^{-1}$ (4.70$\;\times \;10^{-6}$) -	**3.4744$\;\times \;10^{-1}$ (6.74$\;\times \;10^{-5}$)**
	3	12	1.9630$\;\times \;10^{-1}$ (9.65$\;\times \;10^{-4}$) -	1.9766$\;\times \;10^{-1}$ (4.40$\;\times \;10^{-4}$) -	1.8191$\;\times \;10^{-1}$ (2.68$\;\times \;10^{-5}$) -	**1.9972$\;\times \;10^{-1}$ (1.87$\;\times \;10^{-4}$)**
DTLZ6	2	11	**3.4755$\;\times \;10^{-1}$ (3.67$\;\times \;10^{-5}$) =**	3.4755$\;\times \;10^{-1}$ (5.26$\;\times \;10^{-5}$) =	3.4721$\;\times \;10^{-1}$ (1.01$\;\times \;10^{-5}$) -	3.4753$\;\times \;10^{-1}$ (4.45$\;\times \;10^{-5}$)
	3	12	**2.0018$\;\times \;10^{-1}$ (4.09$\;\times \;10^{-5}$) =**	2.0017$\;\times \;10^{-1}$ (4.09$\;\times \;10^{-5}$) =	1.8187$\;\times \;10^{-1}$ (1.12$\;\times \;10^{-4}$) -	2.0017$\;\times \;10^{-1}$ (3.98$\;\times \;10^{-5}$)
DTLZ7	2	21	2.3843$\;\times \;10^{-1}$ (1.70$\;\times \;10^{-2}$) -	2.3839$\;\times \;10^{-1}$ (1.69$\;\times \;10^{-2}$) -	2.2259$\;\times \;10^{-1}$ (2.88$\;\times \;10^{-2}$) -	**2.4294$\;\times \;10^{-1}$ (1.03$\;\times \;10^{-5}$)**
	3	22	2.7138$\;\times \;10^{-1}$ (1.60$\;\times \;10^{-3}$) -	2.6245$\;\times \;10^{-1}$ (1.85$\;\times \;10^{-2}$) -	2.5032$\;\times \;10^{-1}$ (1.32$\;\times \;10^{-2}$) -	**2.7871$\;\times \;10^{-1}$ (5.17$\;\times \;10^{-4}$)**
WFG1	2	11	5.5400$\;\times \;10^{-3}$ (8.56$\;\times \;10^{-3}$) -	3.3397$\;\times \;10^{-1}$ (4.65$\;\times \;10^{-2}$) -	6.2054$\;\times \;10^{-1}$ (1.24$\;\times \;10^{-2}$) +	5.0330$\;\times \;10^{-1}$ (5.46$\;\times \;10^{-2}$)
	3	12	1.0248$\;\times \;10^{-1}$ (2.21$\;\times \;10^{-2}$) -	3.1667$\;\times \;10^{-1}$ (1.98$\;\times \;10^{-2}$) -	8.4128$\;\times \;10^{-1}$ (3.92$\;\times \;10^{-2}$) +	5.6834$\;\times \;10^{-1}$ (5.89$\;\times \;10^{-2}$)
WFG2	2	11	6.3175$\;\times \;10^{-1}$ (3.41$\;\times \;10^{-4}$) =	6.3196$\;\times \;10^{-1}$ (3.95$\;\times \;10^{-4}$) =	6.1218$\;\times \;10^{-1}$ (8.57$\;\times \;10^{-3}$) -	6.1720$\;\times \;10^{-1}$ (3.33$\;\times \;10^{-2}$)
	3	12	**9.2764$\;\times \;10^{-1}$ (1.28$\;\times \;10^{-3}$) +**	9.2689$\;\times \;10^{-1}$ (2.04$\;\times \;10^{-3}$) +	8.9243$\;\times \;10^{-1}$ (1.45$\;\times \;10^{-2}$) -	9.1668$\;\times \;10^{-1}$ (3.63$\;\times \;10^{-2}$)
WFG3	2	11	5.7980$\;\times \;10^{-1}$ (3.01$\;\times \;10^{-4}$) +	5.7966$\;\times \;10^{-1}$ (3.02$\;\times \;10^{-4}$) +	5.7376$\;\times \;10^{-1}$ (2.14$\;\times \;10^{-3}$) +	5.7214$\;\times \;10^{-1}$ (2.70$\;\times \;10^{-2}$)
	3	12	3.2577$\;\times \;10^{-1}$ (6.90$\;\times \;10^{-3}$) -	3.5440$\;\times \;10^{-1}$ (7.68$\;\times \;10^{-3}$) -	3.5180$\;\times \;10^{-1}$ (1.12$\;\times \;10^{-2}$) -	3.6348$\;\times \;10^{-1}$ (3.13$\;\times \;10^{-2}$)
WFG4	2	11	3.1782$\;\times \;10^{-1}$ (1.16$\;\times \;10^{-3}$) -	3.2833$\;\times \;10^{-1}$ (6.54$\;\times \;10^{-3}$) -	3.3684$\;\times \;10^{-1}$ (1.71$\;\times \;10^{-3}$) -	3.3972$\;\times \;10^{-1}$ (3.23$\;\times \;10^{-3}$)
	3	12	4.8848$\;\times \;10^{-1}$ (3.95$\;\times \;10^{-3}$) -	4.9065$\;\times \;10^{-1}$ (3.94$\;\times \;10^{-3}$) -	**5.2865$\;\times \;10^{-1}$ (4.09$\;\times \;10^{-3}$) +**	5.0911$\;\times \;10^{-1}$ (8.68$\;\times \;10^{-3}$)
WFG5	2	11	3.1084$\;\times \;10^{-1}$ (1.58$\;\times \;10^{-3}$) -	3.1021$\;\times \;10^{-1}$ (2.03$\;\times \;10^{-3}$) -	3.0645$\;\times \;10^{-1}$ (6.45$\;\times \;10^{-4}$) -	**3.1363$\;\times \;10^{-1}$ (1.39$\;\times \;10^{-4}$)**
	3	12	4.8677$\;\times \;10^{-1}$ (5.07$\;\times \;10^{-3}$) -	4.8050$\;\times \;10^{-1}$ (5.54$\;\times \;10^{-3}$) -	4.9739$\;\times \;10^{-1}$ (3.17$\;\times \;10^{-3}$) -	**5.0496$\;\times \;10^{-1}$ (3.75$\;\times \;10^{-3}$)**
WFG6	2	11	**3.4199$\;\times \;10^{-1}$ (6.34$\;\times \;10^{-3}$) +**	3.4151$\;\times \;10^{-1}$ (4.22$\;\times \;10^{-3}$) +	2.9466$\;\times \;10^{-1}$ (1.19$\;\times \;10^{-2}$) +	2.2697$\;\times \;10^{-1}$ (5.36$\;\times \;10^{-5}$)
	3	12	**5.2590$\;\times \;10^{-1}$ (5.49$\;\times \;10^{-3}$) +**	5.1572$\;\times \;10^{-1}$ (8.77$\;\times \;10^{-3}$) +	4.7715$\;\times \;10^{-1}$ (1.76$\;\times \;10^{-2}$) +	4.1628$\;\times \;10^{-1}$ (8.87$\;\times \;10^{-4}$)
WFG7	2	11	3.4528$\;\times \;10^{-1}$ (2.57$\;\times \;10^{-4}$) -	3.4525$\;\times \;10^{-1}$ (2.83$\;\times \;10^{-4}$) -	3.3629$\;\times \;10^{-1}$ (1.67$\;\times \;10^{-3}$) -	**3.4719$\;\times \;10^{-1}$ (1.15$\;\times \;10^{-4}$)**
	3	12	5.3049$\;\times \;10^{-1}$ (3.25$\;\times \;10^{-3}$) -	5.2333$\;\times \;10^{-1}$ (3.58$\;\times \;10^{-3}$) -	5.0286$\;\times \;10^{-1}$ (2.07$\;\times \;10^{-2}$) -	**5.5302$\;\times \;10^{-1}$ (2.17$\;\times \;10^{-3}$)**
WFG8	2	11	2.8272$\;\times \;10^{-1}$ (1.61$\;\times \;10^{-3}$) -	2.8459$\;\times \;10^{-1}$ (1.61$\;\times \;10^{-3}$) -	2.7964$\;\times \;10^{-1}$ (4.85$\;\times \;10^{-3}$) -	2.8525$\;\times \;10^{-1}$ (1.69$\;\times \;10^{-3}$)
	3	12	4.2466$\;\times \;10^{-1}$ (5.19$\;\times \;10^{-3}$) -	4.2870$\;\times \;10^{-1}$ (5.08$\;\times \;10^{-3}$) -	**4.5009$\;\times \;10^{-1}$ (6.01$\;\times \;10^{-3}$) +**	4.4087$\;\times \;10^{-1}$ (6.67$\;\times \;10^{-3}$)
WFG9	2	11	3.3360$\;\times \;10^{-1}$ (1.95$\;\times \;10^{-3}$) +	3.3452$\;\times \;10^{-1}$ (1.99$\;\times \;10^{-3}$) +	3.0765$\;\times \;10^{-1}$ (3.14$\;\times \;10^{-2}$) +	2.2643$\;\times \;10^{-1}$ (1.04$\;\times \;10^{-3}$)
	3	12	4.9830$\;\times \;10^{-1}$ (4.16$\;\times \;10^{-2}$) +	**5.1324$\;\times \;10^{-1}$ (4.38$\;\times \;10^{-3}$) +**	4.6505$\;\times \;10^{-1}$ (2.97$\;\times \;10^{-2}$) +	4.0623$\;\times \;10^{-1}$ (2.68$\;\times \;10^{-3}$)
ZDT1	2	30	7.1962$\;\times \;10^{-1}$ (1.51$\;\times \;10^{-4}$) -	7.1928$\;\times \;10^{-1}$ (2.26$\;\times \;10^{-4}$) -	7.0904$\;\times \;10^{-1}$ (9.57$\;\times \;10^{-3}$) -	**7.2049$\;\times \;10^{-1}$ (5.25$\;\times \;10^{-5}$)**
ZDT2	2	30	4.4442$\;\times \;10^{-1}$ (1.64$\;\times \;10^{-4}$) -	4.4401$\;\times \;10^{-1}$ (2.07$\;\times \;10^{-4}$) -	4.0696$\;\times \;10^{-1}$ (4.05$\;\times \;10^{-2}$) -	**4.4510$\;\times \;10^{-1}$ (4.02$\;\times \;10^{-5}$)**
ZDT3	2	30	5.9952$\;\times \;10^{-1}$ (9.36$\;\times \;10^{-5}$) -	5.9965$\;\times \;10^{-1}$ (1.10$\;\times \;10^{-4}$) -	**6.2106$\;\times \;10^{-1}$ (5.00$\;\times \;10^{-2}$) =**	5.9979$\;\times \;10^{-1}$ (2.39$\;\times \;10^{-5}$)
ZDT4	2	10	0.0000$\;\times \;10^{0}$ (0.00$\;\times \;10^{0}$) =	4.4020$\;\times \;10^{-1}$ (2.14$\;\times \;10^{-1}$) +	6.9311$\;\times \;10^{-1}$ (1.65$\;\times \;10^{-2}$) +	0.0000$\;\times \;10^{0}$ (0.00$\;\times \;10^{0}$)
+/-/=			6/22/8	8/22/6	15/19/2	
**Problem**	**M**	**D**	**NSGAII**	**MOEADDE**	**MMOPSO**	**CMOSCA**
DTLZ1	2	6	5.7995$\;\times \;10^{-1}$ (1.60$\;\times \;10^{-3}$) +	**5.8013$\;\times \;10^{-1}$ (4.16$\;\times \;10^{-3}$) +**	5.6631$\;\times \;10^{-1}$ (6.57$\;\times \;10^{-2}$) +	0.0000$\;\times \;10^{0}$ (0.00$\;\times \;10^{0}$)
	3	7	7.9637$\;\times \;10^{-1}$ (1.21$\;\times \;10^{-1}$) +	6.9729$\;\times \;10^{-1}$ (2.31$\;\times \;10^{-1}$) +	2.7383$\;\times \;10^{-1}$ (3.22$\;\times \;10^{-1}$) +	0.0000$\;\times \;10^{0}$ (0.00$\;\times \;10^{0}$)
DTLZ2	2	11	3.4656$\;\times \;10^{-1}$ (2.11$\;\times \;10^{-4}$) -	3.4702$\;\times \;10^{-1}$ (3.78$\;\times \;10^{-5}$) -	3.4668$\;\times \;10^{-1}$ (2.19$\;\times \;10^{-4}$) -	**3.4743$\;\times \;10^{-1}$ (5.68$\;\times \;10^{-5}$)**
	3	12	5.2873$\;\times \;10^{-1}$ (4.91$\;\times \;10^{-3}$) -	5.2691$\;\times \;10^{-1}$ (1.65$\;\times \;10^{-3}$) -	5.3071$\;\times \;10^{-1}$ (4.40$\;\times \;10^{-3}$) -	5.5278$\;\times \;10^{-1}$ (1.94$\;\times \;10^{-3}$)
DTLZ3	2	11	**3.0575$\;\times \;10^{-1}$ (8.42$\;\times \;10^{-2}$) +**	5.6429$\;\times \;10^{-2}$ (1.19$\;\times \;10^{-1}$) +	0.0000$\;\times \;10^{0}$ (0.00$\;\times \;10^{0}$) =	0.0000$\;\times \;10^{0}$ (0.00$\;\times \;10^{0}$)
	3	12	**3.8921$\;\times \;10^{-1}$ (1.62$\;\times \;10^{-1}$) +**	1.8937$\;\times \;10^{-1}$ (2.22$\;\times \;10^{-1}$) +	1.5222$\;\times \;10^{-2}$ (8.34$\;\times \;10^{-2}$) =	0.0000$\;\times \;10^{0}$ (0.00$\;\times \;10^{0}$)
DTLZ4	2	11	3.1245$\;\times \;10^{-1}$ (8.84$\;\times \;10^{-2}$) -	3.4683$\;\times \;10^{-1}$ (6.56$\;\times \;10^{-5}$) -	3.0403$\;\times \;10^{-1}$ (9.70$\;\times \;10^{-2}$) -	**3.4733$\;\times \;10^{-1}$ (7.32$\;\times \;10^{-5}$)**
	3	12	5.1573$\;\times \;10^{-1}$ (8.03$\;\times \;10^{-2}$) -	5.1937$\;\times \;10^{-1}$ (2.29$\;\times \;10^{-2}$) -	5.3269$\;\times \;10^{-1}$ (4.90$\;\times \;10^{-3}$) -	**5.3966$\;\times \;10^{-1}$ (3.31$\;\times \;10^{-3}$)**
DTLZ5	2	11	3.4650$\;\times \;10^{-1}$ (2.07$\;\times \;10^{-4}$) -	3.4701$\;\times \;10^{-1}$ (3.21$\;\times \;10^{-5}$) -	3.4664$\;\times \;10^{-1}$ (1.78$\;\times \;10^{-4}$) -	**3.4744$\;\times \;10^{-1}$ (6.74$\;\times \;10^{-5}$)**
	3	12	1.9874$\;\times \;10^{-1}$ (2.30$\;\times \;10^{-4}$) -	1.9438$\;\times \;10^{-1}$ (7.71$\;\times \;10^{-5}$) -	1.9916$\;\times \;10^{-1}$ (1.77$\;\times \;10^{-4}$) -	**1.9972$\;\times \;10^{-1}$ (1.87$\;\times \;10^{-4}$)**
DTLZ6	2	11	3.4638$\;\times \;10^{-1}$ (2.33$\;\times \;10^{-4}$) -	3.4721$\;\times \;10^{-1}$ (7.87$\;\times \;10^{-8}$) -	3.4663$\;\times \;10^{-1}$ (2.18$\;\times \;10^{-4}$) -	3.4753$\;\times \;10^{-1}$ (4.45$\;\times \;10^{-5}$)
	3	12	1.9912$\;\times \;10^{-1}$ (1.80$\;\times \;10^{-4}$) -	1.9477$\;\times \;10^{-1}$ (2.22$\;\times \;10^{-5}$) -	1.9922$\;\times \;10^{-1}$ (1.59$\;\times \;10^{-4}$) -	2.0017$\;\times \;10^{-1}$ (3.98$\;\times \;10^{-5}$)
DTLZ7	2	21	2.4272$\;\times \;10^{-1}$ (4.13$\;\times \;10^{-5}$) -	2.2855$\;\times \;10^{-1}$ (2.69$\;\times \;10^{-2}$) -	2.1603$\;\times \;10^{-1}$ (3.32$\;\times \;10^{-2}$) -	**2.4294$\;\times \;10^{-1}$ (1.03$\;\times \;10^{-5}$)**
	3	22	2.6689$\;\times \;10^{-1}$ (2.12$\;\times \;10^{-3}$) -	2.0996$\;\times \;10^{-1}$ (1.76$\;\times \;10^{-2}$) -	2.5880$\;\times \;10^{-1}$ (1.52$\;\times \;10^{-2}$) -	**2.7871$\;\times \;10^{-1}$ (5.17$\;\times \;10^{-4}$)**
WFG1	2	11	**6.6884$\;\times \;10^{-1}$ (1.32$\;\times \;10^{-2}$) +**	4.9843$\;\times \;10^{-1}$ (4.87$\;\times \;10^{-2}$) =	6.5123$\;\times \;10^{-1}$ (2.76$\;\times \;10^{-2}$) +	5.0330$\;\times \;10^{-1}$ (5.46$\;\times \;10^{-2}$)
	3	12	**9.2184$\;\times \;10^{-1}$ (5.97$\;\times \;10^{-3}$) +**	4.0981$\;\times \;10^{-1}$ (5.21$\;\times \;10^{-2}$) -	8.1347$\;\times \;10^{-1}$ (2.81$\;\times \;10^{-2}$) +	5.6834$\;\times \;10^{-1}$ (5.89$\;\times \;10^{-2}$)
WFG2	2	11	6.3234$\;\times \;10^{-1}$ (4.78$\;\times \;10^{-4}$) +	6.2802$\;\times \;10^{-1}$ (9.83$\;\times \;10^{-4}$) +	**6.3307$\;\times \;10^{-1}$ (1.90$\;\times \;10^{-4}$) +**	6.1720$\;\times \;10^{-1}$ (3.33$\;\times \;10^{-2}$)
	3	12	9.1559$\;\times \;10^{-1}$ (3.51$\;\times \;10^{-3}$) -	8.7644$\;\times \;10^{-1}$ (6.11$\;\times \;10^{-3}$) -	9.1401$\;\times \;10^{-1}$ (3.42$\;\times \;10^{-3}$) -	9.1668$\;\times \;10^{-1}$ (3.63$\;\times \;10^{-2}$)
WFG3	2	11	5.7929$\;\times \;10^{-1}$ (6.66$\;\times \;10^{-4}$) +	5.7806$\;\times \;10^{-1}$ (6.61$\;\times \;10^{-4}$) +	**5.8024$\;\times \;10^{-1}$ (4.29$\;\times \;10^{-4}$) +**	5.7214$\;\times \;10^{-1}$ (2.70$\;\times \;10^{-2}$)
	3	12	**3.9521$\;\times \;10^{-1}$ (3.69$\;\times \;10^{-3}$) +**	3.4249$\;\times \;10^{-1}$ (1.55$\;\times \;10^{-2}$) -	3.9426$\;\times \;10^{-1}$ (5.41$\;\times \;10^{-3}$) +	3.6348$\;\times \;10^{-1}$ (3.13$\;\times \;10^{-2}$)
WFG4	2	11	**3.4575$\;\times \;10^{-1}$ (2.63$\;\times \;10^{-4}$) +**	3.2351$\;\times \;10^{-1}$ (3.49$\;\times \;10^{-3}$) -	3.4422$\;\times \;10^{-1}$ (6.90$\;\times \;10^{-4}$) +	3.3972$\;\times \;10^{-1}$ (3.23$\;\times \;10^{-3}$)
	3	12	5.1694$\;\times \;10^{-1}$ (6.01$\;\times \;10^{-3}$) +	4.6432$\;\times \;10^{-1}$ (7.04$\;\times \;10^{-3}$) -	4.9689$\;\times \;10^{-1}$ (6.50$\;\times \;10^{-3}$) -	5.0911$\;\times \;10^{-1}$ (8.68$\;\times \;10^{-3}$)
WFG5	2	11	3.1238$\;\times \;10^{-1}$ (1.39$\;\times \;10^{-3}$) -	3.0803$\;\times \;10^{-1}$ (1.69$\;\times \;10^{-3}$) -	3.1289$\;\times \;10^{-1}$ (1.73$\;\times \;10^{-4}$) -	**3.1363$\;\times \;10^{-1}$ (1.39$\;\times \;10^{-4}$)**
	3	12	4.8840$\;\times \;10^{-1}$ (5.07$\;\times \;10^{-3}$) -	4.5556$\;\times \;10^{-1}$ (3.66$\;\times \;10^{-3}$) -	4.8551$\;\times \;10^{-1}$ (7.37$\;\times \;10^{-3}$) -	**5.0496$\;\times \;10^{-1}$ (3.75$\;\times \;10^{-3}$)**
WFG6	2	11	3.0608$\;\times \;10^{-1}$ (1.10$\;\times \;10^{-2}$) +	3.1454$\;\times \;10^{-1}$ (4.13$\;\times \;10^{-2}$) +	3.1757$\;\times \;10^{-1}$ (3.76$\;\times \;10^{-2}$) +	2.2697$\;\times \;10^{-1}$ (5.36$\;\times \;10^{-5}$)
	3	12	4.7001$\;\times \;10^{-1}$ (1.56$\;\times \;10^{-2}$) +	4.2788$\;\times \;10^{-1}$ (4.57$\;\times \;10^{-2}$) +	4.6580$\;\times \;10^{-1}$ (4.83$\;\times \;10^{-2}$) +	4.1628$\;\times \;10^{-1}$ (8.87$\;\times \;10^{-4}$)
WFG7	2	11	3.4503$\;\times \;10^{-1}$ (3.59$\;\times \;10^{-4}$) -	3.4474$\;\times \;10^{-1}$ (2.90$\;\times \;10^{-4}$) -	3.4583$\;\times \;10^{-1}$ (2.75$\;\times \;10^{-4}$) -	**3.4719$\;\times \;10^{-1}$ (1.15$\;\times \;10^{-4}$)**
	3	12	5.1679$\;\times \;10^{-1}$ (4.32$\;\times \;10^{-3}$) -	4.9114$\;\times \;10^{-1}$ (5.76$\;\times \;10^{-3}$) -	5.2276$\;\times \;10^{-1}$ (5.08$\;\times \;10^{-3}$) -	**5.5302$\;\times \;10^{-1}$ (2.17$\;\times \;10^{-3}$)**
WFG8	2	11	2.8775$\;\times \;10^{-1}$ (6.89$\;\times \;10^{-4}$) +	**2.8973$\;\times \;10^{-1}$ (2.51$\;\times \;10^{-3}$) +**	2.8842$\;\times \;10^{-1}$ (1.33$\;\times \;10^{-3}$) +	2.8525$\;\times \;10^{-1}$ (1.69$\;\times \;10^{-3}$)
	3	12	4.3610$\;\times \;10^{-1}$ (4.36$\;\times \;10^{-3}$) -	3.9117$\;\times \;10^{-1}$ (9.68$\;\times \;10^{-3}$) -	4.3457$\;\times \;10^{-1}$ (5.02$\;\times \;10^{-3}$) -	4.4087$\;\times \;10^{-1}$ (6.67$\;\times \;10^{-3}$)
WFG9	2	11	3.3651$\;\times \;10^{-1}$ (2.11$\;\times \;10^{-2}$) +	3.3126$\;\times \;10^{-1}$ (2.28$\;\times \;10^{-3}$) +	**3.3914$\;\times \;10^{-1}$ (2.07$\;\times \;10^{-3}$) +**	2.2643$\;\times \;10^{-1}$ (1.04$\;\times \;10^{-3}$)
	3	12	4.8142$\;\times \;10^{-1}$ (3.13$\;\times \;10^{-2}$) +	4.7633$\;\times \;10^{-1}$ (5.60$\;\times \;10^{-3}$) +	4.9105$\;\times \;10^{-1}$ (2.08$\;\times \;10^{-2}$) +	4.0623$\;\times \;10^{-1}$ (2.68$\;\times \;10^{-3}$)
ZDT1	2	30	7.1910$\;\times \;10^{-1}$ (2.07$\;\times \;10^{-4}$) -	7.0598$\;\times \;10^{-1}$ (4.70$\;\times \;10^{-3}$) -	7.1932$\;\times \;10^{-1}$ (2.26$\;\times \;10^{-4}$) -	**7.2049$\;\times \;10^{-1}$ (5.25$\;\times \;10^{-5}$)**
ZDT2	2	30	4.4383$\;\times \;10^{-1}$ (2.18$\;\times \;10^{-4}$) -	4.3332$\;\times \;10^{-1}$ (3.65$\;\times \;10^{-3}$) -	4.4400$\;\times \;10^{-1}$ (2.21$\;\times \;10^{-4}$) -	**4.4510$\;\times \;10^{-1}$ (4.02$\;\times \;10^{-5}$)**
ZDT3	2	30	6.0228$\;\times \;10^{-1}$ (1.62$\;\times \;10^{-2}$) +	5.9542$\;\times \;10^{-1}$ (6.91$\;\times \;10^{-3}$) -	5.9942$\;\times \;10^{-1}$ (9.92$\;\times \;10^{-5}$) -	5.9979$\;\times \;10^{-1}$ (2.39$\;\times \;10^{-5}$)
ZDT4	2	10	**7.1724$\;\times \;10^{-1}$ (1.44$\;\times \;10^{-3}$) +**	4.8618$\;\times \;10^{-1}$ (1.38$\;\times \;10^{-1}$) +	6.9739$\;\times \;10^{-1}$ (6.24$\;\times \;10^{-2}$) +	0.0000$\;\times \;10^{0}$ (0.00$\;\times \;10^{0}$)
+/-/=			18/18/0	12/23/1	14/20/2	

“+”, “=” and “-” indicate that the results of competing MOEAs are statistically superior, similar, and inferior to results obtained by the CMOSCA, respectively. The best result of each test problem is displayed in bold.

**Table 5:** IGD values achieved by CMOSCA using different β values.

Problem	M	D	CMOSCA β= 2	CMOSCA β=10	CMOSCA β= 15	CMOSCA β= 5
DTLZ1	2	6	1.9342$\;\times \;10^{1}$ (5.45$\;\times \;10^{0}$) =	**1.5759$\;\times \;10^{1}$ (5.60$\;\times \;10^{0}$) =**	1.7927$\;\times \;10^{1}$ (4.60$\;\times \;10^{0}$) =	1.8233$\;\times \;10^{1}$ (6.02$\;\times \;10^{0}$)
	3	7	1.1176$\;\times \;10^{1}$ (3.34$\;\times \;10^{0}$) =	1.0587$\;\times \;10^{1}$ (4.83$\;\times \;10^{0}$) =	1.2098$\;\times \;10^{1}$ (4.92$\;\times \;10^{0}$) =	1.1081$\;\times \;10^{1}$ (4.81$\;\times \;10^{0}$)
DTLZ2	2	11	4.1321$\;\times \;10^{-3}$ (2.54$\;\times \;10^{-5}$) =	4.1045$\;\times \;10^{-3}$ (2.59$\;\times \;10^{-5}$) =	4.1115$\;\times \;10^{-3}$ (3.16$\;\times \;10^{-5}$) =	4.1149$\;\times \;10^{-3}$ (3.78$\;\times \;10^{-5}$)
	3	12	5.3615$\;\times \;10^{-2}$ (6.60$\;\times \;10^{-4}$) -	5.3771$\;\times \;10^{-2}$ (6.50$\;\times \;10^{-4}$) -	5.4166$\;\times \;10^{-2}$ (7.16$\;\times \;10^{-4}$) -	**5.3243$\;\times \;10^{-2}$ (4.56$\;\times \;10^{-4}$)**
DTLZ3	2	11	1.7068$\;\times \;10^{2}$ (1.35$\;\times \;10^{1}$) -	1.6755$\;\times \;10^{2}$ (2.07$\;\times \;10^{1}$) -	1.6684$\;\times \;10^{2}$ (1.54$\;\times \;10^{1}$) -	**1.5902$\;\times \;10^{2}$ (1.94$\;\times \;10^{1}$)**
	3	12	**1.5333$\;\times \;10^{2}$ (1.49$\;\times \;10^{1}$) +**	1.6358$\;\times \;10^{2}$ (1.43$\;\times \;10^{1}$) =	1.6016$\;\times \;10^{2}$ (1.60$\;\times \;10^{1}$) =	1.6203$\;\times \;10^{2}$ (1.60$\;\times \;10^{1}$)
DTLZ4	2	11	4.1405$\;\times \;10^{-3}$ (4.41$\;\times \;10^{-5}$) -	4.1098$\;\times \;10^{-3}$ (2.08$\;\times \;10^{-5}$) =	4.1039$\;\times \;10^{-3}$ (3.59$\;\times \;10^{-5}$) =	4.1119$\;\times \;10^{-3}$ (3.26$\;\times \;10^{-5}$)
	3	12	5.7988$\;\times \;10^{-2}$ (1.69$\;\times \;10^{-3}$) +	5.8669$\;\times \;10^{-2}$ (2.07$\;\times \;10^{-3}$) =	5.8298$\;\times \;10^{-2}$ (1.97$\;\times \;10^{-3}$) +	5.9577$\;\times \;10^{-2}$ (1.47$\;\times \;10^{-3}$)
DTLZ5	2	11	4.1298$\;\times \;10^{-3}$ (2.95$\;\times \;10^{-5}$) -	**4.1037$\;\times \;10^{-3}$ (3.67$\;\times \;10^{-5}$) =**	4.1130$\;\times \;10^{-3}$ (3.83$\;\times \;10^{-5}$) =	4.1107$\;\times \;10^{-3}$ (2.65$\;\times \;10^{-5}$)
	3	12	4.5261$\;\times \;10^{-3}$ (2.47$\;\times \;10^{-4}$) -	**4.3021$\;\times \;10^{-3}$ (1.38$\;\times \;10^{-4}$) =**	4.3323$\;\times \;10^{-3}$ (1.75$\;\times \;10^{-4}$) =	4.3665$\;\times \;10^{-3}$ (1.69$\;\times \;10^{-4}$)
DTLZ6	2	11	4.1265$\;\times \;10^{-3}$ (2.99$\;\times \;10^{-5}$) =	4.1003$\;\times \;10^{-3}$ (3.25$\;\times \;10^{-5}$) =	**4.0799$\;\times \;10^{-3}$ (3.55$\;\times \;10^{-5}$) +**	4.1140$\;\times \;10^{-3}$ (3.03$\;\times \;10^{-5}$)
	3	12	4.2171$\;\times \;10^{-3}$ (8.36$\;\times \;10^{-5}$) =	4.1822$\;\times \;10^{-3}$ (4.75$\;\times \;10^{-5}$) =	4.1835$\;\times \;10^{-3}$ (4.99$\;\times \;10^{-5}$) =	4.1788$\;\times \;10^{-3}$ (5.83$\;\times \;10^{-5}$)
DTLZ7	2	21	4.5212$\;\times \;10^{-3}$ (4.63$\;\times \;10^{-5}$) =	4.5097$\;\times \;10^{-3}$ (6.11$\;\times \;10^{-5}$) =	4.5075$\;\times \;10^{-3}$ (5.73$\;\times \;10^{-5}$) =	4.5057$\;\times \;10^{-3}$ (5.13$\;\times \;10^{-5}$)
	3	22	5.8902$\;\times \;10^{-2}$ (1.13$\;\times \;10^{-3}$) =	5.8975$\;\times \;10^{-2}$ (1.10$\;\times \;10^{-3}$) =	5.8410$\;\times \;10^{-2}$ (8.37$\;\times \;10^{-4}$) +	5.8941$\;\times \;10^{-2}$ (6.68$\;\times \;10^{-4}$)
WFG1	2	11	4.4875$\;\times \;10^{-1}$ (1.15$\;\times \;10^{-1}$) -	3.9312$\;\times \;10^{-1}$ (1.50$\;\times \;10^{-1}$) =	**3.4205$\;\times \;10^{-1}$ (6.73$\;\times \;10^{-2}$) =**	3.8033$\;\times \;10^{-1}$ (1.19$\;\times \;10^{-1}$)
	3	12	**7.0762$\;\times \;10^{-1}$ (1.32$\;\times \;10^{-1}$) +**	1.1179$\;\times \;10^{0}$ (1.55$\;\times \;10^{-1}$) -	1.2398$\;\times \;10^{0}$ (1.14$\;\times \;10^{-1}$) -	8.5394$\;\times \;10^{-1}$ (1.43$\;\times \;10^{-1}$)
WFG2	2	11	3.5297$\;\times \;10^{-2}$ (4.81$\;\times \;10^{-2}$) +	3.1870$\;\times \;10^{-2}$ (4.89$\;\times \;10^{-2}$) =	2.1731$\;\times \;10^{-2}$ (3.61$\;\times \;10^{-2}$) =	3.5635$\;\times \;10^{-2}$ (5.38$\;\times \;10^{-2}$)
	3	12	1.9292$\;\times \;10^{-1}$ (3.11$\;\times \;10^{-2}$) =	1.9270$\;\times \;10^{-1}$ (3.23$\;\times \;10^{-2}$) =	**1.8012$\;\times \;10^{-1}$ (4.91$\;\times \;10^{-3}$) =**	1.9064$\;\times \;10^{-1}$ (3.08$\;\times \;10^{-2}$)
WFG3	2	11	1.0300$\;\times \;10^{-1}$ (9.72$\;\times \;10^{-2}$) -	2.3611$\;\times \;10^{-2}$ (4.27$\;\times \;10^{-2}$) =	1.8118$\;\times \;10^{-2}$ (3.09$\;\times \;10^{-2}$) =	2.9280$\;\times \;10^{-2}$ (5.16$\;\times \;10^{-2}$)
	3	12	1.7046$\;\times \;10^{-1}$ (6.91$\;\times \;10^{-2}$) -	**1.1792$\;\times \;10^{-1}$ (1.20$\;\times \;10^{-2}$) =**	1.2634$\;\times \;10^{-1}$ (3.23$\;\times \;10^{-2}$) =	1.3883$\;\times \;10^{-1}$ (6.04$\;\times \;10^{-2}$)
WFG4	2	11	2.5007$\;\times \;10^{-2}$ (6.96$\;\times \;10^{-3}$) -	2.1590$\;\times \;10^{-2}$ (4.26$\;\times \;10^{-3}$) =	2.1172$\;\times \;10^{-2}$ (5.39$\;\times \;10^{-3}$) =	**2.0532$\;\times \;10^{-2}$ (4.51$\;\times \;10^{-3}$)**
	3	12	**2.4046$\;\times \;10^{-1}$ (1.03$\;\times \;10^{-2}$) =**	2.5109$\;\times \;10^{-1}$ (6.54$\;\times \;10^{-3}$) -	2.5137$\;\times \;10^{-1}$ (4.65$\;\times \;10^{-3}$) -	2.4095$\;\times \;10^{-1}$ (8.39$\;\times \;10^{-3}$)
WFG5	2	11	6.3656$\;\times \;10^{-2}$ (3.17$\;\times \;10^{-4}$) =	6.4203$\;\times \;10^{-2}$ (1.56$\;\times \;10^{-3}$) -	6.5213$\;\times \;10^{-2}$ (2.10$\;\times \;10^{-3}$) -	**6.3608$\;\times \;10^{-2}$ (1.83$\;\times \;10^{-4}$)**
	3	12	2.2368$\;\times \;10^{-1}$ (3.67$\;\times \;10^{-3}$) =	2.2769$\;\times \;10^{-1}$ (6.21$\;\times \;10^{-3}$) -	2.2713$\;\times \;10^{-1}$ (4.49$\;\times \;10^{-3}$) -	**2.2262$\;\times \;10^{-1}$ (3.68$\;\times \;10^{-3}$)**
WFG6	2	11	2.2547$\;\times \;10^{-1}$ (2.73$\;\times \;10^{-5}$) =	2.2547$\;\times \;10^{-1}$ (2.81$\;\times \;10^{-5}$) =	**2.2546$\;\times \;10^{-1}$ (2.27$\;\times \;10^{-5}$) =**	2.2547$\;\times \;10^{-1}$ (1.68$\;\times \;10^{-5}$)
	3	12	3.3557$\;\times \;10^{-1}$ (1.70$\;\times \;10^{-3}$) =	3.3865$\;\times \;10^{-1}$ (5.34$\;\times \;10^{-3}$) -	3.4089$\;\times \;10^{-1}$ (6.47$\;\times \;10^{-3}$) -	**3.3555$\;\times \;10^{-1}$ (1.77$\;\times \;10^{-3}$)**
WFG7	2	11	1.2707$\;\times \;10^{-2}$ (2.23$\;\times \;10^{-4}$) =	1.2798$\;\times \;10^{-2}$ (1.62$\;\times \;10^{-4}$) -	1.2839$\;\times \;10^{-2}$ (1.68$\;\times \;10^{-4}$) -	**1.2639$\;\times \;10^{-2}$ (1.05$\;\times \;10^{-4}$)**
	3	12	**2.1060$\;\times \;10^{-1}$ (2.51$\;\times \;10^{-3}$) =**	2.1305$\;\times \;10^{-1}$ (2.58$\;\times \;10^{-3}$) -	2.1501$\;\times \;10^{-1}$ (3.23$\;\times \;10^{-3}$) -	2.1066$\;\times \;10^{-1}$ (1.88$\;\times \;10^{-3}$)
WFG8	2	11	1.1816$\;\times \;10^{-1}$ (7.35$\;\times \;10^{-3}$) =	1.1589$\;\times \;10^{-1}$ (3.96$\;\times \;10^{-3}$) =	1.1436$\;\times \;10^{-1}$ (2.76$\;\times \;10^{-3}$) +	1.1660$\;\times \;10^{-1}$ (3.17$\;\times \;10^{-3}$)
	3	12	**3.0929$\;\times \;10^{-1}$ (9.22$\;\times \;10^{-3}$) +**	3.1856$\;\times \;10^{-1}$ (6.98$\;\times \;10^{-3}$) =	3.2552$\;\times \;10^{-1}$ (9.20$\;\times \;10^{-3}$) -	3.1606$\;\times \;10^{-1}$ (9.77$\;\times \;10^{-3}$)
WFG9	2	11	**2.1662$\;\times \;10^{-1}$ (3.48$\;\times \;10^{-2}$) +**	2.1669$\;\times \;10^{-1}$ (4.58$\;\times \;10^{-2}$) +	2.2253$\;\times \;10^{-1}$ (3.17$\;\times \;10^{-2}$) +	2.2676$\;\times \;10^{-1}$ (2.47$\;\times \;10^{-3}$)
	3	12	**3.4457$\;\times \;10^{-1}$ (3.72$\;\times \;10^{-3}$) =**	3.4867$\;\times \;10^{-1}$ (5.88$\;\times \;10^{-3}$) -	3.4698$\;\times \;10^{-1}$ (4.62$\;\times \;10^{-3}$) -	3.4464$\;\times \;10^{-1}$ (3.88$\;\times \;10^{-3}$)
ZDT1	2	30	3.9024$\;\times \;10^{-3}$ (3.96$\;\times \;10^{-5}$) =	3.8799$\;\times \;10^{-3}$ (5.26$\;\times \;10^{-5}$) =	3.8795$\;\times \;10^{-3}$ (4.55$\;\times \;10^{-5}$) =	3.8994$\;\times \;10^{-3}$ (4.98$\;\times \;10^{-5}$)
ZDT2	2	30	3.8961$\;\times \;10^{-3}$ (4.41$\;\times \;10^{-5}$) =	3.8674$\;\times \;10^{-3}$ (2.93$\;\times \;10^{-5}$) +	3.8695$\;\times \;10^{-3}$ (4.29$\;\times \;10^{-5}$) +	3.8933$\;\times \;10^{-3}$ (3.48$\;\times \;10^{-5}$)
ZDT3	2	30	**4.6500$\;\times \;10^{-3}$ (4.80$\;\times \;10^{-5}$) +**	4.6965$\;\times \;10^{-3}$ (5.97$\;\times \;10^{-5}$) =	4.6808$\;\times \;10^{-3}$ (6.32$\;\times \;10^{-5}$) =	4.6982$\;\times \;10^{-3}$ (7.19$\;\times \;10^{-5}$)
ZDT4	2	10	2.2937$\;\times \;10^{1}$ (1.94$\;\times \;10^{1}$) =	2.2757$\;\times \;10^{1}$ (2.03$\;\times \;10^{1}$) =	1.9949$\;\times \;10^{1}$ (2.04$\;\times \;10^{1}$) =	1.7501$\;\times \;10^{1}$ (1.63$\;\times \;10^{1}$)
+/-/=			7/9/20	2/10/24	6/11/19	
**Problem**	**M**	**D**	**CMOSCA β= 2**	**CMOSCA β=10**	**CMOSCA β= 15**	**CMOSCA β= 5**
DTLZ1	2	6	1.6761$\;\times \;10^{1}$ (3.94$\;\times \;10^{0}$) =	1.5874$\;\times \;10^{1}$ (3.94$\;\times \;10^{0}$) =	1.7088$\;\times \;10^{1}$ (3.40$\;\times \;10^{0}$) =	1.8233$\;\times \;10^{1}$ (6.02$\;\times \;10^{0}$)
	3	7	1.1537$\;\times \;10^{1}$ (4.01$\;\times \;10^{0}$) =	1.0875$\;\times \;10^{1}$ (4.65$\;\times \;10^{0}$) =	**9.7277$\;\times \;10^{0}$ (3.74$\;\times \;10^{0}$) =**	1.1081$\;\times \;10^{1}$ (4.81$\;\times \;10^{0}$)
DTLZ2	2	11	**4.0992$\;\times \;10^{-3}$ (2.59$\;\times \;10^{-5}$) =**	4.1089$\;\times \;10^{-3}$ (3.28$\;\times \;10^{-5}$) =	4.1011$\;\times \;10^{-3}$ (2.87$\;\times \;10^{-5}$) =	4.1149$\;\times \;10^{-3}$ (3.78$\;\times \;10^{-5}$)
	3	12	5.4058$\;\times \;10^{-2}$ (7.27$\;\times \;10^{-4}$) -	5.4141$\;\times \;10^{-2}$ (6.94$\;\times \;10^{-4}$) -	5.4381$\;\times \;10^{-2}$ (7.65$\;\times \;10^{-4}$) -	**5.3243$\;\times \;10^{-2}$ (4.56$\;\times \;10^{-4}$)**
DTLZ3	2	11	1.6731$\;\times \;10^{2}$ (1.56$\;\times \;10^{1}$) =	1.6652$\;\times \;10^{2}$ (1.58$\;\times \;10^{1}$) =	1.6064$\;\times \;10^{2}$ (1.71$\;\times \;10^{1}$) =	**1.5902$\;\times \;10^{2}$ (1.94$\;\times \;10^{1}$)**
	3	12	1.6290$\;\times \;10^{2}$ (1.42$\;\times \;10^{1}$) =	1.5810$\;\times \;10^{2}$ (1.35$\;\times \;10^{1}$) =	1.6358$\;\times \;10^{2}$ (1.34$\;\times \;10^{1}$) =	1.6203$\;\times \;10^{2}$ (1.60$\;\times \;10^{1}$)
DTLZ4	2	11	**4.1022$\;\times \;10^{-3}$ (3.43$\;\times \;10^{-5}$) =**	4.1235$\;\times \;10^{-3}$ (3.78$\;\times \;10^{-5}$) =	4.1188$\;\times \;10^{-3}$ (3.42$\;\times \;10^{-5}$) =	4.1119$\;\times \;10^{-3}$ (3.26$\;\times \;10^{-5}$)
	3	12	5.8481$\;\times \;10^{-2}$ (2.17$\;\times \;10^{-3}$) +	5.8062$\;\times \;10^{-2}$ (1.63$\;\times \;10^{-3}$) +	**5.7916$\;\times \;10^{-2}$ (1.36$\;\times \;10^{-3}$) +**	5.9577$\;\times \;10^{-2}$ (1.47$\;\times \;10^{-3}$)
DTLZ5	2	11	4.1053$\;\times \;10^{-3}$ (3.52$\;\times \;10^{-5}$) =	4.1058$\;\times \;10^{-3}$ (3.48$\;\times \;10^{-5}$) =	4.1101$\;\times \;10^{-3}$ (2.91$\;\times \;10^{-5}$) =	4.1107$\;\times \;10^{-3}$ (2.65$\;\times \;10^{-5}$)
	3	12	4.3433$\;\times \;10^{-3}$ (2.32$\;\times \;10^{-4}$) =	4.3389$\;\times \;10^{-3}$ (1.90$\;\times \;10^{-4}$) =	4.3671$\;\times \;10^{-3}$ (2.53$\;\times \;10^{-4}$) =	4.3665$\;\times \;10^{-3}$ (1.69$\;\times \;10^{-4}$)
DTLZ6	2	11	4.0799$\;\times \;10^{-3}$ (3.35$\;\times \;10^{-5}$) +	4.0871$\;\times \;10^{-3}$ (2.94$\;\times \;10^{-5}$) +	4.0828$\;\times \;10^{-3}$ (3.18$\;\times \;10^{-5}$) +	4.1140$\;\times \;10^{-3}$ (3.03$\;\times \;10^{-5}$)
	3	12	4.1752$\;\times \;10^{-3}$ (4.29$\;\times \;10^{-5}$) =	**4.1549$\;\times \;10^{-3}$ (3.64$\;\times \;10^{-5}$) +**	4.1770$\;\times \;10^{-3}$ (4.24$\;\times \;10^{-5}$) =	4.1788$\;\times \;10^{-3}$ (5.83$\;\times \;10^{-5}$)
DTLZ7	2	21	4.4963$\;\times \;10^{-3}$ (4.94$\;\times \;10^{-5}$) =	**4.4795$\;\times \;10^{-3}$ (5.82$\;\times \;10^{-5}$) =**	4.4983$\;\times \;10^{-3}$ (5.59$\;\times \;10^{-5}$) =	4.5057$\;\times \;10^{-3}$ (5.13$\;\times \;10^{-5}$)
	3	22	**5.8313$\;\times \;10^{-2}$ (8.07$\;\times \;10^{-4}$) +**	5.8443$\;\times \;10^{-2}$ (1.06$\;\times \;10^{-3}$) +	5.8670$\;\times \;10^{-2}$ (1.23$\;\times \;10^{-3}$) =	5.8941$\;\times \;10^{-2}$ (6.68$\;\times \;10^{-4}$)
WFG1	2	11	3.7226$\;\times \;10^{-1}$ (8.60$\;\times \;10^{-2}$) =	4.5902$\;\times \;10^{-1}$ (8.82$\;\times \;10^{-2}$) -	4.3078$\;\times \;10^{-1}$ (5.81$\;\times \;10^{-2}$) -	3.8033$\;\times \;10^{-1}$ (1.19$\;\times \;10^{-1}$)
	3	12	1.2694$\;\times \;10^{0}$ (1.05$\;\times \;10^{-1}$) -	1.2971$\;\times \;10^{0}$ (9.35$\;\times \;10^{-2}$) -	1.3041$\;\times \;10^{0}$ (1.05$\;\times \;10^{-1}$) -	8.5394$\;\times \;10^{-1}$ (1.43$\;\times \;10^{-1}$)
WFG2	2	11	2.1843$\;\times \;10^{-2}$ (3.60$\;\times \;10^{-2}$) =	**1.7349$\;\times \;10^{-2}$ (2.59$\;\times \;10^{-2}$) +**	2.1994$\;\times \;10^{-2}$ (3.63$\;\times \;10^{-2}$) =	3.5635$\;\times \;10^{-2}$ (5.38$\;\times \;10^{-2}$)
	3	12	1.8193$\;\times \;10^{-1}$ (4.51$\;\times \;10^{-3}$) =	1.8330$\;\times \;10^{-1}$ (1.89$\;\times \;10^{-2}$) =	1.8331$\;\times \;10^{-1}$ (7.77$\;\times \;10^{-3}$) =	1.9064$\;\times \;10^{-1}$ (3.08$\;\times \;10^{-2}$)
WFG3	2	11	**1.2539$\;\times \;10^{-2}$ (3.20$\;\times \;10^{-4}$) =**	1.8201$\;\times \;10^{-2}$ (3.10$\;\times \;10^{-2}$) =	1.8254$\;\times \;10^{-2}$ (3.08$\;\times \;10^{-2}$) +	2.9280$\;\times \;10^{-2}$ (5.16$\;\times \;10^{-2}$)
	3	12	1.2889$\;\times \;10^{-1}$ (9.48$\;\times \;10^{-3}$) +	1.3009$\;\times \;10^{-1}$ (1.47$\;\times \;10^{-2}$) +	1.3413$\;\times \;10^{-1}$ (1.38$\;\times \;10^{-2}$) +	1.3883$\;\times \;10^{-1}$ (6.04$\;\times \;10^{-2}$)
WFG4	2	11	2.4712$\;\times \;10^{-2}$ (8.76$\;\times \;10^{-3}$) =	2.3005$\;\times \;10^{-2}$ (6.39$\;\times \;10^{-3}$) =	2.7480$\;\times \;10^{-2}$ (8.18$\;\times \;10^{-3}$) -	**2.0532$\;\times \;10^{-2}$ (4.51$\;\times \;10^{-3}$)**
	3	12	2.5300$\;\times \;10^{-1}$ (4.36$\;\times \;10^{-3}$) -	2.5269$\;\times \;10^{-1}$ (5.29$\;\times \;10^{-3}$) -	2.5231$\;\times \;10^{-1}$ (4.47$\;\times \;10^{-3}$) -	2.4095$\;\times \;10^{-1}$ (8.39$\;\times \;10^{-3}$)
WFG5	2	11	6.5284$\;\times \;10^{-2}$ (2.41$\;\times \;10^{-3}$) -	6.5157$\;\times \;10^{-2}$ (2.16$\;\times \;10^{-3}$) -	6.6438$\;\times \;10^{-2}$ (2.55$\;\times \;10^{-3}$) -	**6.3608$\;\times \;10^{-2}$ (1.83$\;\times \;10^{-4}$)**
	3	12	2.2939$\;\times \;10^{-1}$ (7.01$\;\times \;10^{-3}$) -	2.3148$\;\times \;10^{-1}$ (6.98$\;\times \;10^{-3}$) -	2.3074$\;\times \;10^{-1}$ (6.44$\;\times \;10^{-3}$) -	**2.2262$\;\times \;10^{-1}$ (3.68$\;\times \;10^{-3}$)**
WFG6	2	11	2.2547$\;\times \;10^{-1}$ (2.02$\;\times \;10^{-5}$) =	2.2547$\;\times \;10^{-1}$ (2.90$\;\times \;10^{-5}$) =	2.2547$\;\times \;10^{-1}$ (3.34$\;\times \;10^{-5}$) =	2.2547$\;\times \;10^{-1}$ (1.68$\;\times \;10^{-5}$)
	3	12	3.4413$\;\times \;10^{-1}$ (1.00$\;\times \;10^{-2}$) -	3.4564$\;\times \;10^{-1}$ (1.06$\;\times \;10^{-2}$) -	3.4362$\;\times \;10^{-1}$ (6.62$\;\times \;10^{-3}$) -	**3.3555$\;\times \;10^{-1}$ (1.77$\;\times \;10^{-3}$)**
WFG7	2	11	1.2856$\;\times \;10^{-2}$ (1.60$\;\times \;10^{-4}$) -	1.2914$\;\times \;10^{-2}$ (1.83$\;\times \;10^{-4}$) -	1.2925$\;\times \;10^{-2}$ (1.68$\;\times \;10^{-4}$) -	**1.2639$\;\times \;10^{-2}$ (1.05$\;\times \;10^{-4}$)**
	3	12	2.1524$\;\times \;10^{-1}$ (2.53$\;\times \;10^{-3}$) -	2.1690$\;\times \;10^{-1}$ (2.85$\;\times \;10^{-3}$) -	2.1856$\;\times \;10^{-1}$ (3.63$\;\times \;10^{-3}$) -	2.1066$\;\times \;10^{-1}$ (1.88$\;\times \;10^{-3}$)
WFG8	2	11	1.1472$\;\times \;10^{-1}$ (2.69$\;\times \;10^{-3}$) +	**1.1336$\;\times \;10^{-1}$ (1.18$\;\times \;10^{-3}$) +**	1.1368$\;\times \;10^{-1}$ (2.55$\;\times \;10^{-3}$) +	1.1660$\;\times \;10^{-1}$ (3.17$\;\times \;10^{-3}$)
	3	12	3.2575$\;\times \;10^{-1}$ (7.52$\;\times \;10^{-3}$) -	3.2585$\;\times \;10^{-1}$ (6.85$\;\times \;10^{-3}$) -	3.2562$\;\times \;10^{-1}$ (8.46$\;\times \;10^{-3}$) -	3.1606$\;\times \;10^{-1}$ (9.77$\;\times \;10^{-3}$)
WFG9	2	11	2.2603$\;\times \;10^{-1}$ (1.26$\;\times \;10^{-2}$) +	2.2263$\;\times \;10^{-1}$ (3.45$\;\times \;10^{-2}$) +	2.2985$\;\times \;10^{-1}$ (3.06$\;\times \;10^{-3}$) -	2.2676$\;\times \;10^{-1}$ (2.47$\;\times \;10^{-3}$)
	3	12	3.4966$\;\times \;10^{-1}$ (4.88$\;\times \;10^{-3}$) -	3.4850$\;\times \;10^{-1}$ (3.19$\;\times \;10^{-3}$) -	3.4840$\;\times \;10^{-1}$ (3.40$\;\times \;10^{-3}$) -	3.4464$\;\times \;10^{-1}$ (3.88$\;\times \;10^{-3}$)
ZDT1	2	30	3.8759$\;\times \;10^{-3}$ (4.42$\;\times \;10^{-5}$) =	3.8755$\;\times \;10^{-3}$ (3.30$\;\times \;10^{-5}$) =	**3.8648$\;\times \;10^{-3}$ (4.20$\;\times \;10^{-5}$) +**	3.8994$\;\times \;10^{-3}$ (4.98$\;\times \;10^{-5}$)
ZDT2	2	30	**3.8570$\;\times \;10^{-3}$ (2.88$\;\times \;10^{-5}$) +**	3.8829$\;\times \;10^{-3}$ (3.72$\;\times \;10^{-5}$) =	3.8800$\;\times \;10^{-3}$ (4.25$\;\times \;10^{-5}$) =	3.8933$\;\times \;10^{-3}$ (3.48$\;\times \;10^{-5}$)
ZDT3	2	30	4.6941$\;\times \;10^{-3}$ (5.65$\;\times \;10^{-5}$) =	4.6906$\;\times \;10^{-3}$ (7.38$\;\times \;10^{-5}$) =	4.6678$\;\times \;10^{-3}$ (6.23$\;\times \;10^{-5}$) =	4.6982$\;\times \;10^{-3}$ (7.19$\;\times \;10^{-5}$)
ZDT4	2	10	**1.0998$\;\times \;10^{1}$ (1.03$\;\times \;10^{1}$) +**	1.7827$\;\times \;10^{1}$ (1.64$\;\times \;10^{1}$) =	1.4417$\;\times \;10^{1}$ (1.35$\;\times \;10^{1}$) =	1.7501$\;\times \;10^{1}$ (1.63$\;\times \;10^{1}$)
+/-/=			8/10/18	8/11/17	6/13/17	

“+”, “=” and “-” indicate that the results of competing CMOSCA using different *β* values are statistically superior, similar, and inferior to results obtained by the CMOSCA using *β* = 5 values, respectively. The best result of each test problem is displayed in bold.

**Table 6:** HV values achieved by CMOSCA using different β values.

Problem	M	D	CMOSCA β= 2	CMOSCA β= 10	CMOSCA β= 15	CMOSCA β= 5
DTLZ1	2	6	0.0000$\;\times \;10^{0}$ (0.00$\;\times \;10^{0}$) =	0.0000$\;\times \;10^{0}$ (0.00$\;\times \;10^{0}$) =	0.0000$\;\times \;10^{0}$ (0.00$\;\times \;10^{0}$) =	0.0000$\;\times \;10^{0}$ (0.00$\;\times \;10^{0}$)
	3	7	0.0000$\;\times \;10^{0}$ (0.00$\;\times \;10^{0}$) =	0.0000$\;\times \;10^{0}$ (0.00$\;\times \;10^{0}$) =	**8.1450$\;\times \;10^{-6}$ (4.46$\;\times \;10^{-5}$) =**	0.0000$\;\times \;10^{0}$ (0.00$\;\times \;10^{0}$)
DTLZ2	2	11	**3.4752$\;\times \;10^{-1}$ (4.20$\;\times \;10^{-5}$) +**	3.4736$\;\times \;10^{-1}$ (7.13$\;\times \;10^{-5}$) -	3.4732$\;\times \;10^{-1}$ (7.84$\;\times \;10^{-5}$) -	3.4743$\;\times \;10^{-1}$ (5.68$\;\times \;10^{-5}$)
	3	12	5.5109$\;\times \;10^{-1}$ (2.45$\;\times \;10^{-3}$) -	5.5078$\;\times \;10^{-1}$ (1.82$\;\times \;10^{-3}$) -	5.4892$\;\times \;10^{-1}$ (2.29$\;\times \;10^{-3}$) -	**5.5278$\;\times \;10^{-1}$ (1.94$\;\times \;10^{-3}$)**
DTLZ3	2	11	0.0000$\;\times \;10^{0}$ (0.00$\;\times \;10^{0}$) =	0.0000$\;\times \;10^{0}$ (0.00$\;\times \;10^{0}$) =	0.0000$\;\times \;10^{0}$ (0.00$\;\times \;10^{0}$) =	0.0000$\;\times \;10^{0}$ (0.00$\;\times \;10^{0}$)
	3	12	0.0000$\;\times \;10^{0}$ (0.00$\;\times \;10^{0}$) =	0.0000$\;\times \;10^{0}$ (0.00$\;\times \;10^{0}$) =	0.0000$\;\times \;10^{0}$ (0.00$\;\times \;10^{0}$) =	0.0000$\;\times \;10^{0}$ (0.00$\;\times \;10^{0}$)
DTLZ4	2	11	3.4728$\;\times \;10^{-1}$ (1.31$\;\times \;10^{-4}$) =	3.4725$\;\times \;10^{-1}$ (8.88$\;\times \;10^{-5}$) -	3.4720$\;\times \;10^{-1}$ (7.92$\;\times \;10^{-5}$) -	**3.4733$\;\times \;10^{-1}$ (7.32$\;\times \;10^{-5}$)**
	3	12	**5.4209$\;\times \;10^{-1}$ (3.87$\;\times \;10^{-3}$) +**	5.4082$\;\times \;10^{-1}$ (4.38$\;\times \;10^{-3}$) =	5.4194$\;\times \;10^{-1}$ (3.67$\;\times \;10^{-3}$) +	5.3966$\;\times \;10^{-1}$ (3.31$\;\times \;10^{-3}$)
DTLZ5	2	11	**3.4753$\;\times \;10^{-1}$ (4.47$\;\times \;10^{-5}$) +**	3.4736$\;\times \;10^{-1}$ (6.34$\;\times \;10^{-5}$) -	3.4730$\;\times \;10^{-1}$ (6.47$\;\times \;10^{-5}$) -	3.4744$\;\times \;10^{-1}$ (6.74$\;\times \;10^{-5}$)
	3	12	1.9949$\;\times \;10^{-1}$ (2.54$\;\times \;10^{-4}$) -	1.9971$\;\times \;10^{-1}$ (1.72$\;\times \;10^{-4}$) =	1.9971$\;\times \;10^{-1}$ (1.63$\;\times \;10^{-4}$) =	**1.9972$\;\times \;10^{-1}$ (1.87$\;\times \;10^{-4}$)**
DTLZ6	2	11	**3.4754$\;\times \;10^{-1}$ (4.62$\;\times \;10^{-5}$) =**	3.4752$\;\times \;10^{-1}$ (5.84$\;\times \;10^{-5}$) =	3.4753$\;\times \;10^{-1}$ (4.14$\;\times \;10^{-5}$) =	3.4753$\;\times \;10^{-1}$ (4.45$\;\times \;10^{-5}$)
	3	12	2.0018$\;\times \;10^{-1}$ (5.24$\;\times \;10^{-5}$) =	2.0018$\;\times \;10^{-1}$ (2.72$\;\times \;10^{-5}$) =	2.0017$\;\times \;10^{-1}$ (3.78$\;\times \;10^{-5}$) =	2.0017$\;\times \;10^{-1}$ (3.98$\;\times \;10^{-5}$)
DTLZ7	2	21	2.4294$\;\times \;10^{-1}$ (9.08$\;\times \;10^{-6}$) =	2.4294$\;\times \;10^{-1}$ (9.57$\;\times \;10^{-6}$) =	2.4294$\;\times \;10^{-1}$ (1.21$\;\times \;10^{-5}$) =	2.4294$\;\times \;10^{-1}$ (1.03$\;\times \;10^{-5}$)
	3	22	2.7867$\;\times \;10^{-1}$ (8.10$\;\times \;10^{-4}$) =	2.7869$\;\times \;10^{-1}$ (7.01$\;\times \;10^{-4}$) =	2.7870$\;\times \;10^{-1}$ (7.05$\;\times \;10^{-4}$) =	**2.7871$\;\times \;10^{-1}$ (5.17$\;\times \;10^{-4}$)**
WFG1	2	11	4.7170$\;\times \;10^{-1}$ (5.12$\;\times \;10^{-2}$) -	4.8977$\;\times \;10^{-1}$ (6.80$\;\times \;10^{-2}$) =	**5.1255$\;\times \;10^{-1}$ (3.70$\;\times \;10^{-2}$) =**	5.0330$\;\times \;10^{-1}$ (5.46$\;\times \;10^{-2}$)
	3	12	**6.2541$\;\times \;10^{-1}$ (6.57$\;\times \;10^{-2}$) +**	4.4784$\;\times \;10^{-1}$ (6.09$\;\times \;10^{-2}$) -	3.9320$\;\times \;10^{-1}$ (3.77$\;\times \;10^{-2}$) -	5.6834$\;\times \;10^{-1}$ (5.89$\;\times \;10^{-2}$)
WFG2	2	11	6.1647$\;\times \;10^{-1}$ (2.97$\;\times \;10^{-2}$) -	6.1919$\;\times \;10^{-1}$ (3.02$\;\times \;10^{-2}$) =	6.2561$\;\times \;10^{-1}$ (2.23$\;\times \;10^{-2}$) =	6.1720$\;\times \;10^{-1}$ (3.33$\;\times \;10^{-2}$)
	3	12	9.1747$\;\times \;10^{-1}$ (3.67$\;\times \;10^{-2}$) =	9.1371$\;\times \;10^{-1}$ (3.63$\;\times \;10^{-2}$) -	**9.2543$\;\times \;10^{-1}$ (1.76$\;\times \;10^{-3}$) +**	9.1668$\;\times \;10^{-1}$ (3.63$\;\times \;10^{-2}$)
WFG3	2	11	5.3376$\;\times \;10^{-1}$ (5.01$\;\times \;10^{-2}$) -	5.7511$\;\times \;10^{-1}$ (2.24$\;\times \;10^{-2}$) =	5.7790$\;\times \;10^{-1}$ (1.61$\;\times \;10^{-2}$) =	5.7214$\;\times \;10^{-1}$ (2.70$\;\times \;10^{-2}$)
	3	12	3.4594$\;\times \;10^{-1}$ (3.59$\;\times \;10^{-2}$) -	**3.7349$\;\times \;10^{-1}$ (7.44$\;\times \;10^{-3}$) =**	3.6804$\;\times \;10^{-1}$ (1.69$\;\times \;10^{-2}$) =	3.6348$\;\times \;10^{-1}$ (3.13$\;\times \;10^{-2}$)
WFG4	2	11	3.3661$\;\times \;10^{-1}$ (4.56$\;\times \;10^{-3}$) -	3.3898$\;\times \;10^{-1}$ (2.94$\;\times \;10^{-3}$) =	3.3972$\;\times \;10^{-1}$ (3.14$\;\times \;10^{-3}$) =	**3.3972$\;\times \;10^{-1}$ (3.23$\;\times \;10^{-3}$)**
	3	12	**5.1155$\;\times \;10^{-1}$ (9.66$\;\times \;10^{-3}$) =**	4.9935$\;\times \;10^{-1}$ (5.02$\;\times \;10^{-3}$) -	4.9652$\;\times \;10^{-1}$ (4.61$\;\times \;10^{-3}$) -	5.0911$\;\times \;10^{-1}$ (8.68$\;\times \;10^{-3}$)
WFG5	2	11	**3.1363$\;\times \;10^{-1}$ (1.93$\;\times \;10^{-4}$) =**	3.1304$\;\times \;10^{-1}$ (1.43$\;\times \;10^{-3}$) -	3.1202$\;\times \;10^{-1}$ (1.98$\;\times \;10^{-3}$) -	3.1363$\;\times \;10^{-1}$ (1.39$\;\times \;10^{-4}$)
	3	12	**5.0575$\;\times \;10^{-1}$ (4.61$\;\times \;10^{-3}$) =**	5.0120$\;\times \;10^{-1}$ (5.25$\;\times \;10^{-3}$) -	5.0072$\;\times \;10^{-1}$ (3.72$\;\times \;10^{-3}$) -	5.0496$\;\times \;10^{-1}$ (3.75$\;\times \;10^{-3}$)
WFG6	2	11	**2.2699$\;\times \;10^{-1}$ (5.14$\;\times \;10^{-5}$) =**	2.2697$\;\times \;10^{-1}$ (5.25$\;\times \;10^{-5}$) =	2.2699$\;\times \;10^{-1}$ (4.78$\;\times \;10^{-5}$) =	2.2697$\;\times \;10^{-1}$ (5.36$\;\times \;10^{-5}$)
	3	12	**4.1675$\;\times \;10^{-1}$ (7.97$\;\times \;10^{-4}$) +**	4.1517$\;\times \;10^{-1}$ (2.78$\;\times \;10^{-3}$) =	4.1348$\;\times \;10^{-1}$ (3.54$\;\times \;10^{-3}$) -	4.1628$\;\times \;10^{-1}$ (8.87$\;\times \;10^{-4}$)
WFG7	2	11	**3.4734$\;\times \;10^{-1}$ (9.62$\;\times \;10^{-5}$) +**	3.4688$\;\times \;10^{-1}$ (1.27$\;\times \;10^{-4}$) -	3.4670$\;\times \;10^{-1}$ (1.31$\;\times \;10^{-4}$) -	3.4719$\;\times \;10^{-1}$ (1.15$\;\times \;10^{-4}$)
	3	12	**5.5486$\;\times \;10^{-1}$ (1.92$\;\times \;10^{-3}$) +**	5.4486$\;\times \;10^{-1}$ (2.45$\;\times \;10^{-3}$) -	5.4097$\;\times \;10^{-1}$ (3.47$\;\times \;10^{-3}$) -	5.5302$\;\times \;10^{-1}$ (2.17$\;\times \;10^{-3}$)
WFG8	2	11	2.8421$\;\times \;10^{-1}$ (3.93$\;\times \;10^{-3}$) =	2.8558$\;\times \;10^{-1}$ (1.94$\;\times \;10^{-3}$) =	2.8630$\;\times \;10^{-1}$ (1.36$\;\times \;10^{-3}$) +	2.8525$\;\times \;10^{-1}$ (1.69$\;\times \;10^{-3}$)
	3	12	**4.4568$\;\times \;10^{-1}$ (6.95$\;\times \;10^{-3}$) +**	4.3986$\;\times \;10^{-1}$ (5.26$\;\times \;10^{-3}$) =	4.3361$\;\times \;10^{-1}$ (6.77$\;\times \;10^{-3}$) -	4.4087$\;\times \;10^{-1}$ (6.67$\;\times \;10^{-3}$)
WFG9	2	11	**2.3244$\;\times \;10^{-1}$ (1.93$\;\times \;10^{-2}$) +**	2.3192$\;\times \;10^{-1}$ (2.41$\;\times \;10^{-2}$) +	2.2874$\;\times \;10^{-1}$ (1.65$\;\times \;10^{-2}$) +	2.2643$\;\times \;10^{-1}$ (1.04$\;\times \;10^{-3}$)
	3	12	**4.0653$\;\times \;10^{-1}$ (2.88$\;\times \;10^{-3}$) =**	4.0391$\;\times \;10^{-1}$ (3.56$\;\times \;10^{-3}$) -	4.0433$\;\times \;10^{-1}$ (2.39$\;\times \;10^{-3}$) -	4.0623$\;\times \;10^{-1}$ (2.68$\;\times \;10^{-3}$)
ZDT1	2	30	**7.2054$\;\times \;10^{-1}$ (2.95$\;\times \;10^{-5}$) +**	7.2050$\;\times \;10^{-1}$ (6.05$\;\times \;10^{-5}$) =	7.2048$\;\times \;10^{-1}$ (6.05$\;\times \;10^{-5}$) =	7.2049$\;\times \;10^{-1}$ (5.25$\;\times \;10^{-5}$)
ZDT2	2	30	4.4509$\;\times \;10^{-1}$ (3.58$\;\times \;10^{-5}$) =	**4.4510$\;\times \;10^{-1}$ (2.96$\;\times \;10^{-5}$) =**	4.4507$\;\times \;10^{-1}$ (3.64$\;\times \;10^{-5}$) -	4.4510$\;\times \;10^{-1}$ (4.02$\;\times \;10^{-5}$)
ZDT3	2	30	**5.9981$\;\times \;10^{-1}$ (1.98$\;\times \;10^{-5}$) +**	5.9979$\;\times \;10^{-1}$ (2.13$\;\times \;10^{-5}$) =	5.9979$\;\times \;10^{-1}$ (2.38$\;\times \;10^{-5}$) =	5.9979$\;\times \;10^{-1}$ (2.39$\;\times \;10^{-5}$)
ZDT4	2	10	0.0000$\;\times \;10^{0}$ (0.00$\;\times \;10^{0}$) =	0.0000$\;\times \;10^{0}$ (0.00$\;\times \;10^{0}$) =	0.0000$\;\times \;10^{0}$ (0.00$\;\times \;10^{0}$) =	0.0000$\;\times \;10^{0}$ (0.00$\;\times \;10^{0}$)
+/-/=			11/7/18	1/12/23	4/14/18	
**Problem**	**M**	**D**	**CMOSCA β= 2**	**CMOSCA β= 10**	**CMOSCA β= 15**	**CMOSCA β= 5**
DTLZ1	2	6	0.0000$\;\times \;10^{0}$ (0.00$\;\times \;10^{0}$) =	0.0000$\;\times \;10^{0}$ (0.00$\;\times \;10^{0}$) =	0.0000$\;\times \;10^{0}$ (0.00$\;\times \;10^{0}$) =	0.0000$\;\times \;10^{0}$ (0.00$\;\times \;10^{0}$)
	3	7	0.0000$\;\times \;10^{0}$ (0.00$\;\times \;10^{0}$) =	0.0000$\;\times \;10^{0}$ (0.00$\;\times \;10^{0}$) =	0.0000$\;\times \;10^{0}$ (0.00$\;\times \;10^{0}$) =	0.0000$\;\times \;10^{0}$ (0.00$\;\times \;10^{0}$)
DTLZ2	2	11	3.4729$\;\times \;10^{-1}$ (8.35$\;\times \;10^{-5}$) -	3.4728$\;\times \;10^{-1}$ (7.07$\;\times \;10^{-5}$) -	3.4727$\;\times \;10^{-1}$ (8.73$\;\times \;10^{-5}$) -	3.4743$\;\times \;10^{-1}$ (5.68$\;\times \;10^{-5}$)
	3	12	5.4915$\;\times \;10^{-1}$ (1.81$\;\times \;10^{-3}$) -	5.4899$\;\times \;10^{-1}$ (2.12$\;\times \;10^{-3}$) -	5.4782$\;\times \;10^{-1}$ (2.21$\;\times \;10^{-3}$) -	**5.5278$\;\times \;10^{-1}$ (1.94$\;\times \;10^{-3}$)**
DTLZ3	2	11	0.0000$\;\times \;10^{0}$ (0.00$\;\times \;10^{0}$) =	0.0000$\;\times \;10^{0}$ (0.00$\;\times \;10^{0}$) =	0.0000$\;\times \;10^{0}$ (0.00$\;\times \;10^{0}$) =	0.0000$\;\times \;10^{0}$ (0.00$\;\times \;10^{0}$)
	3	12	0.0000$\;\times \;10^{0}$ (0.00$\;\times \;10^{0}$) =	0.0000$\;\times \;10^{0}$ (0.00$\;\times \;10^{0}$) =	0.0000$\;\times \;10^{0}$ (0.00$\;\times \;10^{0}$) =	0.0000$\;\times \;10^{0}$ (0.00$\;\times \;10^{0}$)
DTLZ4	2	11	3.4719$\;\times \;10^{-1}$ (1.08$\;\times \;10^{-4}$) -	3.4718$\;\times \;10^{-1}$ (1.25$\;\times \;10^{-4}$) -	3.4719$\;\times \;10^{-1}$ (9.34$\;\times \;10^{-5}$) -	**3.4733$\;\times \;10^{-1}$ (7.32$\;\times \;10^{-5}$)**
	3	12	5.4133$\;\times \;10^{-1}$ (4.38$\;\times \;10^{-3}$) +	5.4152$\;\times \;10^{-1}$ (3.51$\;\times \;10^{-3}$) +	5.4120$\;\times \;10^{-1}$ (3.08$\;\times \;10^{-3}$) =	5.3966$\;\times \;10^{-1}$ (3.31$\;\times \;10^{-3}$)
DTLZ5	2	11	3.4731$\;\times \;10^{-1}$ (7.37$\;\times \;10^{-5}$) -	3.4727$\;\times \;10^{-1}$ (7.93$\;\times \;10^{-5}$) -	3.4724$\;\times \;10^{-1}$ (7.99$\;\times \;10^{-5}$) -	3.4744$\;\times \;10^{-1}$ (6.74$\;\times \;10^{-5}$)
	3	12	1.9967$\;\times \;10^{-1}$ (1.92$\;\times \;10^{-4}$) =	1.9968$\;\times \;10^{-1}$ (1.78$\;\times \;10^{-4}$) =	1.9963$\;\times \;10^{-1}$ (2.07$\;\times \;10^{-4}$) =	**1.9972$\;\times \;10^{-1}$ (1.87$\;\times \;10^{-4}$)**
DTLZ6	2	11	3.4754$\;\times \;10^{-1}$ (4.09$\;\times \;10^{-5}$) =	3.4754$\;\times \;10^{-1}$ (4.31$\;\times \;10^{-5}$) =	3.4753$\;\times \;10^{-1}$ (4.83$\;\times \;10^{-5}$) =	3.4753$\;\times \;10^{-1}$ (4.45$\;\times \;10^{-5}$)
	3	12	2.0018$\;\times \;10^{-1}$ (4.17$\;\times \;10^{-5}$) =	2.0016$\;\times \;10^{-1}$ (3.80$\;\times \;10^{-5}$) =	**2.0019$\;\times \;10^{-1}$ (3.24$\;\times \;10^{-5}$) =**	2.0017$\;\times \;10^{-1}$ (3.98$\;\times \;10^{-5}$)
DTLZ7	2	21	2.4294$\;\times \;10^{-1}$ (1.09$\;\times \;10^{-5}$) =	**2.4295$\;\times \;10^{-1}$ (1.23$\;\times \;10^{-5}$) =**	2.4295$\;\times \;10^{-1}$ (1.08$\;\times \;10^{-5}$) =	2.4294$\;\times \;10^{-1}$ (1.03$\;\times \;10^{-5}$)
	3	22	2.7868$\;\times \;10^{-1}$ (7.45$\;\times \;10^{-4}$) =	2.7842$\;\times \;10^{-1}$ (8.96$\;\times \;10^{-4}$) =	2.7850$\;\times \;10^{-1}$ (5.86$\;\times \;10^{-4}$) =	**2.7871$\;\times \;10^{-1}$ (5.17$\;\times \;10^{-4}$)**
WFG1	2	11	4.9539$\;\times \;10^{-1}$ (4.76$\;\times \;10^{-2}$) =	4.4960$\;\times \;10^{-1}$ (4.55$\;\times \;10^{-2}$) -	4.6903$\;\times \;10^{-1}$ (2.90$\;\times \;10^{-2}$) -	5.0330$\;\times \;10^{-1}$ (5.46$\;\times \;10^{-2}$)
	3	12	3.7740$\;\times \;10^{-1}$ (3.59$\;\times \;10^{-2}$) -	3.6193$\;\times \;10^{-1}$ (3.29$\;\times \;10^{-2}$) -	3.5814$\;\times \;10^{-1}$ (3.51$\;\times \;10^{-2}$) -	5.6834$\;\times \;10^{-1}$ (5.89$\;\times \;10^{-2}$)
WFG2	2	11	6.2556$\;\times \;10^{-1}$ (2.23$\;\times \;10^{-2}$) =	**6.2816$\;\times \;10^{-1}$ (1.60$\;\times \;10^{-2}$) +**	6.2541$\;\times \;10^{-1}$ (2.24$\;\times \;10^{-2}$) =	6.1720$\;\times \;10^{-1}$ (3.33$\;\times \;10^{-2}$)
	3	12	9.2445$\;\times \;10^{-1}$ (2.07$\;\times \;10^{-3}$) +	9.2069$\;\times \;10^{-1}$ (1.90$\;\times \;10^{-2}$) +	9.2197$\;\times \;10^{-1}$ (2.12$\;\times \;10^{-3}$) +	9.1668$\;\times \;10^{-1}$ (3.63$\;\times \;10^{-2}$)
WFG3	2	11	**5.8079$\;\times \;10^{-1}$ (3.54$\;\times \;10^{-4}$) =**	5.7781$\;\times \;10^{-1}$ (1.62$\;\times \;10^{-2}$) =	5.7776$\;\times \;10^{-1}$ (1.61$\;\times \;10^{-2}$) +	5.7214$\;\times \;10^{-1}$ (2.70$\;\times \;10^{-2}$)
	3	12	3.6678$\;\times \;10^{-1}$ (6.44$\;\times \;10^{-3}$) +	3.6538$\;\times \;10^{-1}$ (7.94$\;\times \;10^{-3}$) +	3.6401$\;\times \;10^{-1}$ (7.27$\;\times \;10^{-3}$) +	3.6348$\;\times \;10^{-1}$ (3.13$\;\times \;10^{-2}$)
WFG4	2	11	3.3771$\;\times \;10^{-1}$ (4.69$\;\times \;10^{-3}$) =	3.3886$\;\times \;10^{-1}$ (3.29$\;\times \;10^{-3}$) =	3.3634$\;\times \;10^{-1}$ (4.43$\;\times \;10^{-3}$) -	**3.3972$\;\times \;10^{-1}$ (3.23$\;\times \;10^{-3}$)**
	3	12	4.9573$\;\times \;10^{-1}$ (3.69$\;\times \;10^{-3}$) -	4.9622$\;\times \;10^{-1}$ (4.49$\;\times \;10^{-3}$) -	4.9613$\;\times \;10^{-1}$ (3.74$\;\times \;10^{-3}$) -	5.0911$\;\times \;10^{-1}$ (8.68$\;\times \;10^{-3}$)
WFG5	2	11	3.1203$\;\times \;10^{-1}$ (2.09$\;\times \;10^{-3}$) -	3.1198$\;\times \;10^{-1}$ (1.94$\;\times \;10^{-3}$) -	3.1099$\;\times \;10^{-1}$ (2.19$\;\times \;10^{-3}$) -	3.1363$\;\times \;10^{-1}$ (1.39$\;\times \;10^{-4}$)
	3	12	4.9873$\;\times \;10^{-1}$ (6.00$\;\times \;10^{-3}$) -	4.9789$\;\times \;10^{-1}$ (5.59$\;\times \;10^{-3}$) -	4.9782$\;\times \;10^{-1}$ (6.11$\;\times \;10^{-3}$) -	5.0496$\;\times \;10^{-1}$ (3.75$\;\times \;10^{-3}$)
WFG6	2	11	2.2697$\;\times \;10^{-1}$ (4.73$\;\times \;10^{-5}$) =	2.2699$\;\times \;10^{-1}$ (4.88$\;\times \;10^{-5}$) +	2.2698$\;\times \;10^{-1}$ (4.77$\;\times \;10^{-5}$) =	2.2697$\;\times \;10^{-1}$ (5.36$\;\times \;10^{-5}$)
	3	12	4.1189$\;\times \;10^{-1}$ (5.26$\;\times \;10^{-3}$) -	4.1146$\;\times \;10^{-1}$ (6.09$\;\times \;10^{-3}$) -	4.1248$\;\times \;10^{-1}$ (3.92$\;\times \;10^{-3}$) -	4.1628$\;\times \;10^{-1}$ (8.87$\;\times \;10^{-4}$)
WFG7	2	11	3.4662$\;\times \;10^{-1}$ (1.46$\;\times \;10^{-4}$) -	3.4660$\;\times \;10^{-1}$ (1.60$\;\times \;10^{-4}$) -	3.4657$\;\times \;10^{-1}$ (1.79$\;\times \;10^{-4}$) -	3.4719$\;\times \;10^{-1}$ (1.15$\;\times \;10^{-4}$)
	3	12	5.3768$\;\times \;10^{-1}$ (3.58$\;\times \;10^{-3}$) -	5.3586$\;\times \;10^{-1}$ (2.44$\;\times \;10^{-3}$) -	5.3436$\;\times \;10^{-1}$ (3.19$\;\times \;10^{-3}$) -	5.5302$\;\times \;10^{-1}$ (2.17$\;\times \;10^{-3}$)
WFG8	2	11	2.8623$\;\times \;10^{-1}$ (1.22$\;\times \;10^{-3}$) +	**2.8674$\;\times \;10^{-1}$ (5.45$\;\times \;10^{-4}$) +**	2.8665$\;\times \;10^{-1}$ (1.19$\;\times \;10^{-3}$) +	2.8525$\;\times \;10^{-1}$ (1.69$\;\times \;10^{-3}$)
	3	12	4.3398$\;\times \;10^{-1}$ (4.56$\;\times \;10^{-3}$) -	4.3449$\;\times \;10^{-1}$ (4.51$\;\times \;10^{-3}$) -	4.3398$\;\times \;10^{-1}$ (6.53$\;\times \;10^{-3}$) -	4.4087$\;\times \;10^{-1}$ (6.67$\;\times \;10^{-3}$)
WFG9	2	11	2.2721$\;\times \;10^{-1}$ (8.25$\;\times \;10^{-3}$) +	2.2889$\;\times \;10^{-1}$ (1.86$\;\times \;10^{-2}$) +	2.2512$\;\times \;10^{-1}$ (1.20$\;\times \;10^{-3}$) -	2.2643$\;\times \;10^{-1}$ (1.04$\;\times \;10^{-3}$)
	3	12	4.0290$\;\times \;10^{-1}$ (2.87$\;\times \;10^{-3}$) -	4.0390$\;\times \;10^{-1}$ (2.62$\;\times \;10^{-3}$) -	4.0374$\;\times \;10^{-1}$ (2.82$\;\times \;10^{-3}$) -	4.0623$\;\times \;10^{-1}$ (2.68$\;\times \;10^{-3}$)
ZDT1	2	30	7.2048$\;\times \;10^{-1}$ (7.51$\;\times \;10^{-5}$) =	7.2049$\;\times \;10^{-1}$ (4.55$\;\times \;10^{-5}$) =	7.2047$\;\times \;10^{-1}$ (4.25$\;\times \;10^{-5}$) =	7.2049$\;\times \;10^{-1}$ (5.25$\;\times \;10^{-5}$)
ZDT2	2	30	4.4509$\;\times \;10^{-1}$ (3.48$\;\times \;10^{-5}$) =	4.4507$\;\times \;10^{-1}$ (4.90$\;\times \;10^{-5}$) -	4.4507$\;\times \;10^{-1}$ (4.96$\;\times \;10^{-5}$) -	4.4510$\;\times \;10^{-1}$ (4.02$\;\times \;10^{-5}$)
ZDT3	2	30	5.9979$\;\times \;10^{-1}$ (2.23$\;\times \;10^{-5}$) =	5.9979$\;\times \;10^{-1}$ (2.81$\;\times \;10^{-5}$) =	5.9980$\;\times \;10^{-1}$ (2.39$\;\times \;10^{-5}$) =	5.9979$\;\times \;10^{-1}$ (2.39$\;\times \;10^{-5}$)
ZDT4	2	10	0.0000$\;\times \;10^{0}$ (0.00$\;\times \;10^{0}$) =	0.0000$\;\times \;10^{0}$ (0.00$\;\times \;10^{0}$) =	0.0000$\;\times \;10^{0}$ (0.00$\;\times \;10^{0}$) =	0.0000$\;\times \;10^{0}$ (0.00$\;\times \;10^{0}$)
+/-/=			5/13/18	7/15/14	4/17/15	

“+”, “=” and “-” indicate that the results of competing CMOSCA using different *β* values are statistically superior, similar, and inferior to results obtained by the CMOSCA using *β* = 5 values, respectively. The best result of each test problem is displayed in bold.

**Table 7:** IGD values achieved by three CMOSCA variants.

Problem	M	D	CMOSCAA	CMOSCAD	CMOSCA
DTLZ1	2	6	1.8997$\;\times \;10^{1}$ (6.26$\;\times \;10^{0}$) =	1.8496$\;\times \;10^{1}$ (6.55$\;\times \;10^{0}$) =	**1.8233$\;\times \;10^{1}$ (6.02$\;\times \;10^{0}$)**
	3	7	1.1280$\;\times \;10^{1}$ (4.33$\;\times \;10^{0}$) =	**1.0678$\;\times \;10^{1}$ (4.45$\;\times \;10^{0}$) =**	1.1081$\;\times \;10^{1}$ (4.81$\;\times \;10^{0}$)
DTLZ2	2	11	**4.1038$\;\times \;10^{-3}$ (3.45$\;\times \;10^{-5}$) =**	4.1125$\;\times \;10^{-3}$ (3.84$\;\times \;10^{-5}$) =	4.1149$\;\times \;10^{-3}$ (3.78$\;\times \;10^{-5}$)
	3	12	5.3713$\;\times \;10^{-2}$ (5.98$\;\times \;10^{-4}$) -	**5.2984$\;\times \;10^{-2}$ (5.50$\;\times \;10^{-4}$) =**	5.3243$\;\times \;10^{-2}$ (4.56$\;\times \;10^{-4}$)
DTLZ3	2	11	1.6913$\;\times \;10^{2}$ (1.87$\;\times \;10^{1}$) -	1.7087$\;\times \;10^{2}$ (1.80$\;\times \;10^{1}$) -	**1.5902$\;\times \;10^{2}$ (1.94$\;\times \;10^{1}$)**
	3	12	**1.5527$\;\times \;10^{2}$ (1.72$\;\times \;10^{1}$) =**	1.6192$\;\times \;10^{2}$ (1.65$\;\times \;10^{1}$) =	1.6203$\;\times \;10^{2}$ (1.60$\;\times \;10^{1}$)
DTLZ4	2	11	4.1188$\;\times \;10^{-3}$ (3.66$\;\times \;10^{-5}$) =	4.1171$\;\times \;10^{-3}$ (3.60$\;\times \;10^{-5}$) =	**4.1119$\;\times \;10^{-3}$ (3.26$\;\times \;10^{-5}$)**
	3	12	5.9014$\;\times \;10^{-2}$ (1.80$\;\times \;10^{-3}$) =	**5.7189$\;\times \;10^{-2}$ (1.96$\;\times \;10^{-3}$) +**	5.9577$\;\times \;10^{-2}$ (1.47$\;\times \;10^{-3}$)
DTLZ5	2	11	**4.1033$\;\times \;10^{-3}$ (4.02$\;\times \;10^{-5}$) =**	4.1142$\;\times \;10^{-3}$ (2.93$\;\times \;10^{-5}$) =	4.1107$\;\times \;10^{-3}$ (2.65$\;\times \;10^{-5}$)
	3	12	5.1442$\;\times \;10^{-3}$ (5.49$\;\times \;10^{-4}$) -	**4.2607$\;\times \;10^{-3}$ (1.03$\;\times \;10^{-4}$) +**	4.3665$\;\times \;10^{-3}$ (1.69$\;\times \;10^{-4}$)
DTLZ6	2	11	**4.0768$\;\times \;10^{-3}$ (2.82$\;\times \;10^{-5}$) +**	4.0938$\;\times \;10^{-3}$ (2.60$\;\times \;10^{-5}$) +	4.1140$\;\times \;10^{-3}$ (3.03$\;\times \;10^{-5}$)
	3	12	**4.1680$\;\times \;10^{-3}$ (5.58$\;\times \;10^{-5}$) =**	4.1686$\;\times \;10^{-3}$ (4.40$\;\times \;10^{-5}$) =	4.1788$\;\times \;10^{-3}$ (5.83$\;\times \;10^{-5}$)
DTLZ7	2	21	**4.4714$\;\times \;10^{-3}$ (5.49$\;\times \;10^{-5}$) +**	4.5119$\;\times \;10^{-3}$ (7.67$\;\times \;10^{-5}$) =	4.5057$\;\times \;10^{-3}$ (5.13$\;\times \;10^{-5}$)
	3	22	**5.8436$\;\times \;10^{-2}$ (1.33$\;\times \;10^{-3}$) +**	5.8680$\;\times \;10^{-2}$ (1.23$\;\times \;10^{-3}$) =	5.8941$\;\times \;10^{-2}$ (6.68$\;\times \;10^{-4}$)
WFG1	2	11	**3.6703$\;\times \;10^{-1}$ (1.09$\;\times \;10^{-1}$) =**	5.7172$\;\times \;10^{-1}$ (1.42$\;\times \;10^{-1}$) -	3.8033$\;\times \;10^{-1}$ (1.19$\;\times \;10^{-1}$)
	3	12	1.1237$\;\times \;10^{0}$ (1.18$\;\times \;10^{-1}$) -	1.3241$\;\times \;10^{0}$ (1.15$\;\times \;10^{-1}$) -	**8.5394$\;\times \;10^{-1}$ (1.43$\;\times \;10^{-1}$)**
WFG2	2	11	4.5567$\;\times \;10^{-2}$ (6.09$\;\times \;10^{-2}$) =	7.4616$\;\times \;10^{-2}$ (7.14$\;\times \;10^{-2}$) -	**3.5635$\;\times \;10^{-2}$ (5.38$\;\times \;10^{-2}$)**
	3	12	**1.8933$\;\times \;10^{-1}$ (2.77$\;\times \;10^{-2}$) =**	2.1603$\;\times \;10^{-1}$ (4.28$\;\times \;10^{-2}$) -	1.9064$\;\times \;10^{-1}$ (3.08$\;\times \;10^{-2}$)
WFG3	2	11	2.9572$\;\times \;10^{-2}$ (5.16$\;\times \;10^{-2}$) -	4.0745$\;\times \;10^{-2}$ (6.41$\;\times \;10^{-2}$) -	**2.9280$\;\times \;10^{-2}$ (5.16$\;\times \;10^{-2}$)**
	3	12	1.4767$\;\times \;10^{-1}$ (2.29$\;\times \;10^{-2}$) -	1.6052$\;\times \;10^{-1}$ (6.33$\;\times \;10^{-2}$) -	**1.3883$\;\times \;10^{-1}$ (6.04$\;\times \;10^{-2}$)**
WFG4	2	11	2.4884$\;\times \;10^{-2}$ (7.53$\;\times \;10^{-3}$) -	2.5662$\;\times \;10^{-2}$ (9.48$\;\times \;10^{-3}$) -	**2.0532$\;\times \;10^{-2}$ (4.51$\;\times \;10^{-3}$)**
	3	12	2.5274$\;\times \;10^{-1}$ (4.80$\;\times \;10^{-3}$) -	2.4420$\;\times \;10^{-1}$ (8.85$\;\times \;10^{-3}$) =	**2.4095$\;\times \;10^{-1}$ (8.39$\;\times \;10^{-3}$)**
WFG5	2	11	6.4533$\;\times \;10^{-2}$ (1.39$\;\times \;10^{-3}$) -	6.3745$\;\times \;10^{-2}$ (1.06$\;\times \;10^{-3}$) =	**6.3608$\;\times \;10^{-2}$ (1.83$\;\times \;10^{-4}$)**
	3	12	2.2664$\;\times \;10^{-1}$ (6.26$\;\times \;10^{-3}$) -	2.2360$\;\times \;10^{-1}$ (4.37$\;\times \;10^{-3}$) =	**2.2262$\;\times \;10^{-1}$ (3.68$\;\times \;10^{-3}$)**
WFG6	2	11	2.2547$\;\times \;10^{-1}$ (5.73$\;\times \;10^{-5}$) -	2.2547$\;\times \;10^{-1}$ (2.02$\;\times \;10^{-5}$) =	**2.2547$\;\times \;10^{-1}$ (1.68$\;\times \;10^{-5}$)**
	3	12	3.3692$\;\times \;10^{-1}$ (5.75$\;\times \;10^{-3}$) =	3.3754$\;\times \;10^{-1}$ (4.19$\;\times \;10^{-3}$) -	**3.3555$\;\times \;10^{-1}$ (1.77$\;\times \;10^{-3}$)**
WFG7	2	11	1.2834$\;\times \;10^{-2}$ (1.42$\;\times \;10^{-4}$) -	1.2805$\;\times \;10^{-2}$ (1.88$\;\times \;10^{-4}$) -	**1.2639$\;\times \;10^{-2}$ (1.05$\;\times \;10^{-4}$)**
	3	12	2.1510$\;\times \;10^{-1}$ (4.05$\;\times \;10^{-3}$) -	2.1444$\;\times \;10^{-1}$ (3.11$\;\times \;10^{-3}$) -	**2.1066$\;\times \;10^{-1}$ (1.88$\;\times \;10^{-3}$)**
WFG8	2	11	**1.1509$\;\times \;10^{-1}$ (3.53$\;\times \;10^{-3}$) +**	1.1843$\;\times \;10^{-1}$ (4.11$\;\times \;10^{-3}$) -	1.1660$\;\times \;10^{-1}$ (3.17$\;\times \;10^{-3}$)
	3	12	3.2616$\;\times \;10^{-1}$ (8.27$\;\times \;10^{-3}$) -	3.2911$\;\times \;10^{-1}$ (1.08$\;\times \;10^{-2}$) -	**3.1606$\;\times \;10^{-1}$ (9.77$\;\times \;10^{-3}$)**
WFG9	2	11	2.2322$\;\times \;10^{-1}$ (2.19$\;\times \;10^{-2}$) +	**2.2021$\;\times \;10^{-1}$ (3.29$\;\times \;10^{-2}$) =**	2.2676$\;\times \;10^{-1}$ (2.47$\;\times \;10^{-3}$)
	3	12	3.4681$\;\times \;10^{-1}$ (4.54$\;\times \;10^{-3}$) =	3.4470$\;\times \;10^{-1}$ (3.49$\;\times \;10^{-3}$) =	**3.4464$\;\times \;10^{-1}$ (3.88$\;\times \;10^{-3}$)**
ZDT1	2	30	**3.8606$\;\times \;10^{-3}$ (4.58$\;\times \;10^{-5}$) +**	3.8777$\;\times \;10^{-3}$ (3.78$\;\times \;10^{-5}$) =	3.8994$\;\times \;10^{-3}$ (4.98$\;\times \;10^{-5}$)
ZDT2	2	30	**3.8505$\;\times \;10^{-3}$ (2.87$\;\times \;10^{-5}$) +**	3.8635$\;\times \;10^{-3}$ (2.90$\;\times \;10^{-5}$) +	3.8933$\;\times \;10^{-3}$ (3.48$\;\times \;10^{-5}$)
ZDT3	2	30	**4.6639$\;\times \;10^{-3}$ (5.03$\;\times \;10^{-5}$) =**	4.6779$\;\times \;10^{-3}$ (4.28$\;\times \;10^{-5}$) =	4.6982$\;\times \;10^{-3}$ (7.19$\;\times \;10^{-5}$)
ZDT4	2	10	2.1828$\;\times \;10^{1}$ (1.56$\;\times \;10^{1}$) =	2.2784$\;\times \;10^{1}$ (1.82$\;\times \;10^{1}$) =	**1.7501$\;\times \;10^{1}$ (1.63$\;\times \;10^{1}$)**
+/-/=			7/14/15	4/13/19	

“+”, “=” and “-” indicate that the results of the CMOSCAA and CMOSCAD are statistically superior, similar, and inferior to results obtained by the CMOSCA, respectively. The best result of each test problem is displayed in bold.

**Table 8:** HV values achieved by three CMOSCA variants.

Problem	M	D	CMOSCAA	CMOSCAD	CMOSCA
DTLZ1	2	6	**4.9801$\;\times \;10^{-4}$ (2.73$\;\times \;10^{-3}$) =**	0.0000$\;\times \;10^{0}$ (0.00$\;\times \;10^{0}$) =	0.0000$\;\times \;10^{0}$ (0.00$\;\times \;10^{0}$)
	3	7	**0.0000$\;\times \;10^{0}$ (0.00$\;\times \;10^{0}$) =**	0.0000$\;\times \;10^{0}$ (0.00$\;\times \;10^{0}$) =	0.0000$\;\times \;10^{0}$ (0.00$\;\times \;10^{0}$)
DTLZ2	2	11	3.4724$\;\times \;10^{-1}$ (9.67$\;\times \;10^{-5}$) -	**3.4750$\;\times \;10^{-1}$ (5.17$\;\times \;10^{-5}$) +**	3.4743$\;\times \;10^{-1}$ (5.68$\;\times \;10^{-5}$)
	3	12	5.5033$\;\times \;10^{-1}$ (2.21$\;\times \;10^{-3}$) -	**5.5478$\;\times \;10^{-1}$ (1.42$\;\times \;10^{-3}$) +**	5.5278$\;\times \;10^{-1}$ (1.94$\;\times \;10^{-3}$)
DTLZ3	2	11	**0.0000$\;\times \;10^{0}$ (0.00$\;\times \;10^{0}$) =**	0.0000$\;\times \;10^{0}$ (0.00$\;\times \;10^{0}$) =	0.0000$\;\times \;10^{0}$ (0.00$\;\times \;10^{0}$)
	3	12	**0.0000$\;\times \;10^{0}$ (0.00$\;\times \;10^{0}$) =**	0.0000$\;\times \;10^{0}$ (0.00$\;\times \;10^{0}$) =	0.0000$\;\times \;10^{0}$ (0.00$\;\times \;10^{0}$)
DTLZ4	2	11	3.4714$\;\times \;10^{-1}$ (7.74$\;\times \;10^{-5}$) -	**3.4743$\;\times \;10^{-1}$ (5.31$\;\times \;10^{-5}$) +**	3.4733$\;\times \;10^{-1}$ (7.32$\;\times \;10^{-5}$)
	3	12	5.4179$\;\times \;10^{-1}$ (3.26$\;\times \;10^{-3}$) +	**5.4538$\;\times \;10^{-1}$ (4.14$\;\times \;10^{-3}$) +**	5.3966$\;\times \;10^{-1}$ (3.31$\;\times \;10^{-3}$)
DTLZ5	2	11	3.4720$\;\times \;10^{-1}$ (8.66$\;\times \;10^{-5}$) -	**3.4750$\;\times \;10^{-1}$ (4.72$\;\times \;10^{-5}$) +**	3.4744$\;\times \;10^{-1}$ (6.74$\;\times \;10^{-5}$)
	3	12	1.9911$\;\times \;10^{-1}$ (3.17$\;\times \;10^{-4}$) -	**1.9987$\;\times \;10^{-1}$ (1.15$\;\times \;10^{-4}$) +**	1.9972$\;\times \;10^{-1}$ (1.87$\;\times \;10^{-4}$)
DTLZ6	2	11	3.4753$\;\times \;10^{-1}$ (4.55$\;\times \;10^{-5}$) =	3.4752$\;\times \;10^{-1}$ (4.51$\;\times \;10^{-5}$) =	**3.4753$\;\times \;10^{-1}$ (4.45$\;\times \;10^{-5}$)**
	3	12	**2.0018$\;\times \;10^{-1}$ (4.58$\;\times \;10^{-5}$) =**	2.0016$\;\times \;10^{-1}$ (2.65$\;\times \;10^{-5}$) =	2.0017$\;\times \;10^{-1}$ (3.98$\;\times \;10^{-5}$)
DTLZ7	2	21	**2.4295$\;\times \;10^{-1}$ (9.49$\;\times \;10^{-6}$) +**	2.4294$\;\times \;10^{-1}$ (1.57$\;\times \;10^{-5}$) =	2.4294$\;\times \;10^{-1}$ (1.03$\;\times \;10^{-5}$)
	3	22	2.7884$\;\times \;10^{-1}$ (7.48$\;\times \;10^{-4}$) =	**2.7891$\;\times \;10^{-1}$ (7.43$\;\times \;10^{-4}$) =**	2.7871$\;\times \;10^{-1}$ (5.17$\;\times \;10^{-4}$)
WFG1	2	11	**5.1069$\;\times \;10^{-1}$ (4.98$\;\times \;10^{-2}$) =**	3.9481$\;\times \;10^{-1}$ (6.46$\;\times \;10^{-2}$) -	5.0330$\;\times \;10^{-1}$ (5.46$\;\times \;10^{-2}$)
	3	12	4.6057$\;\times \;10^{-1}$ (5.11$\;\times \;10^{-2}$) -	3.6556$\;\times \;10^{-1}$ (5.26$\;\times \;10^{-2}$) -	**5.6834$\;\times \;10^{-1}$ (5.89$\;\times \;10^{-2}$)**
WFG2	2	11	6.1075$\;\times \;10^{-1}$ (3.76$\;\times \;10^{-2}$) =	5.9275$\;\times \;10^{-1}$ (4.40$\;\times \;10^{-2}$) -	**6.1720$\;\times \;10^{-1}$ (3.33$\;\times \;10^{-2}$)**
	3	12	**9.1862$\;\times \;10^{-1}$ (3.17$\;\times \;10^{-2}$) +**	8.8812$\;\times \;10^{-1}$ (5.24$\;\times \;10^{-2}$) -	9.1668$\;\times \;10^{-1}$ (3.63$\;\times \;10^{-2}$)
WFG3	2	11	5.7178$\;\times \;10^{-1}$ (2.69$\;\times \;10^{-2}$) -	5.6606$\;\times \;10^{-1}$ (3.36$\;\times \;10^{-2}$) -	**5.7214$\;\times \;10^{-1}$ (2.70$\;\times \;10^{-2}$)**
	3	12	3.5253$\;\times \;10^{-1}$ (1.39$\;\times \;10^{-2}$) -	3.5365$\;\times \;10^{-1}$ (3.06$\;\times \;10^{-2}$) -	**3.6348$\;\times \;10^{-1}$ (3.13$\;\times \;10^{-2}$)**
WFG4	2	11	3.3711$\;\times \;10^{-1}$ (4.72$\;\times \;10^{-3}$) -	3.3624$\;\times \;10^{-1}$ (5.95$\;\times \;10^{-3}$) -	**3.3972$\;\times \;10^{-1}$ (3.23$\;\times \;10^{-3}$)**
	3	12	4.9800$\;\times \;10^{-1}$ (4.03$\;\times \;10^{-3}$) -	5.0639$\;\times \;10^{-1}$ (8.82$\;\times \;10^{-3}$) =	**5.0911$\;\times \;10^{-1}$ (8.68$\;\times \;10^{-3}$)**
WFG5	2	11	3.1259$\;\times \;10^{-1}$ (1.39$\;\times \;10^{-3}$) -	3.1344$\;\times \;10^{-1}$ (9.88$\;\times \;10^{-4}$) =	**3.1363$\;\times \;10^{-1}$ (1.39$\;\times \;10^{-4}$)**
	3	12	5.0278$\;\times \;10^{-1}$ (4.50$\;\times \;10^{-3}$) -	**5.0607$\;\times \;10^{-1}$ (4.39$\;\times \;10^{-3}$) =**	5.0496$\;\times \;10^{-1}$ (3.75$\;\times \;10^{-3}$)
WFG6	2	11	2.2696$\;\times \;10^{-1}$ (5.03$\;\times \;10^{-5}$) =	**2.2697$\;\times \;10^{-1}$ (6.21$\;\times \;10^{-5}$) =**	2.2697$\;\times \;10^{-1}$ (5.36$\;\times \;10^{-5}$)
	3	12	4.1606$\;\times \;10^{-1}$ (3.21$\;\times \;10^{-3}$) =	4.1608$\;\times \;10^{-1}$ (2.25$\;\times \;10^{-3}$) =	**4.1628$\;\times \;10^{-1}$ (8.87$\;\times \;10^{-4}$)**
WFG7	2	11	3.4660$\;\times \;10^{-1}$ (1.39$\;\times \;10^{-4}$) -	3.4695$\;\times \;10^{-1}$ (1.13$\;\times \;10^{-4}$) -	**3.4719$\;\times \;10^{-1}$ (1.15$\;\times \;10^{-4}$)**
	3	12	5.4076$\;\times \;10^{-1}$ (4.01$\;\times \;10^{-3}$) -	5.4761$\;\times \;10^{-1}$ (3.09$\;\times \;10^{-3}$) -	**5.5302$\;\times \;10^{-1}$ (2.17$\;\times \;10^{-3}$)**
WFG8	2	11	**2.8598$\;\times \;10^{-1}$ (1.86$\;\times \;10^{-3}$) +**	2.8431$\;\times \;10^{-1}$ (2.18$\;\times \;10^{-3}$) =	2.8525$\;\times \;10^{-1}$ (1.69$\;\times \;10^{-3}$)
	3	12	4.3333$\;\times \;10^{-1}$ (6.61$\;\times \;10^{-3}$) -	4.3261$\;\times \;10^{-1}$ (7.31$\;\times \;10^{-3}$) -	**4.4087$\;\times \;10^{-1}$ (6.67$\;\times \;10^{-3}$)**
WFG9	2	11	2.2865$\;\times \;10^{-1}$ (1.23$\;\times \;10^{-2}$) +	**2.2982$\;\times \;10^{-1}$ (1.74$\;\times \;10^{-2}$) =**	2.2643$\;\times \;10^{-1}$ (1.04$\;\times \;10^{-3}$)
	3	12	4.0447$\;\times \;10^{-1}$ (2.41$\;\times \;10^{-3}$) -	4.0602$\;\times \;10^{-1}$ (2.33$\;\times \;10^{-3}$) =	**4.0623$\;\times \;10^{-1}$ (2.68$\;\times \;10^{-3}$)**
ZDT1	2	30	**7.2055$\;\times \;10^{-1}$ (3.46$\;\times \;10^{-5}$) +**	7.2051$\;\times \;10^{-1}$ (4.45$\;\times \;10^{-5}$) =	7.2049$\;\times \;10^{-1}$ (5.25$\;\times \;10^{-5}$)
ZDT2	2	30	**4.4511$\;\times \;10^{-1}$ (2.69$\;\times \;10^{-5}$) =**	4.4508$\;\times \;10^{-1}$ (3.60$\;\times \;10^{-5}$) -	4.4510$\;\times \;10^{-1}$ (4.02$\;\times \;10^{-5}$)
ZDT3	2	30	**5.9980$\;\times \;10^{-1}$ (2.09$\;\times \;10^{-5}$) =**	5.9980$\;\times \;10^{-1}$ (1.98$\;\times \;10^{-5}$) =	5.9979$\;\times \;10^{-1}$ (2.39$\;\times \;10^{-5}$)
ZDT4	2	10	**0.0000$\;\times \;10^{0}$ (0.00$\;\times \;10^{0}$) =**	0.0000$\;\times \;10^{0}$ (0.00$\;\times \;10^{0}$) =	0.0000$\;\times \;10^{0}$ (0.00$\;\times \;10^{0}$)
+/-/=			6/16/14	6/11/19	

“+”, “=” and “-” indicate that the results of the CMOSCAA and CMOSCAD are statistically superior, similar, and inferior to results obtained by the CMOSCA, respectively. The best result of each test problem is displayed in bold.

**Table 9:** HV results obtained by the seven competing MOEAs on engineering design problems.

Problem	M	D	EMOSO	CMOPSO	MOEAD	CMOSCA
Four bar truss design	2	4	5.9810$\;\times \;10^{-1}$ (1.24$\;\times \;10^{-9}$) -	5.9810$\;\times \;10^{-1}$ (1.09$\;\times \;10^{-9}$) -	5.9810$\;\times \;10^{-1}$ (8.25$\;\times \;10^{-10}$) -	**5.9810$\;\times \;10^{-1}$ (9.02$\;\times \;10^{-10}$)**
Hatch cover design	2	2	**8.8750$\;\times \;10^{-1}$ (5.76$\;\times \;10^{-6}$) =**	8.8750$\;\times \;10^{-1}$ (5.49$\;\times \;10^{-6}$) =	8.7980$\;\times \;10^{-1}$ (5.37$\;\times \;10^{-5}$) -	8.8750$\;\times \;10^{-1}$ (5.81$\;\times \;10^{-6}$)
Two bar truss design	3	3	9.8210$\;\times \;10^{-1}$ (6.34$\;\times \;10^{-3}$) =	9.8187$\;\times \;10^{-1}$ (8.00$\;\times \;10^{-3}$) =	9.8748$\;\times \;10^{-1}$ (2.99$\;\times \;10^{-6}$) =	9.8546$\;\times \;10^{-1}$ (7.64$\;\times \;10^{-3}$)
Welded beam design	3	4	7.8936$\;\times \;10^{-1}$ (4.04$\;\times \;10^{-1}$) =	7.5242$\;\times \;10^{-1}$ (4.25$\;\times \;10^{-1}$) =	9.9990$\;\times \;10^{-1}$ (1.55$\;\times \;10^{-5}$) +	6.0661$\;\times \;10^{-1}$ (4.60$\;\times \;10^{-1}$)
Vehicle crashworthiness design	3	5	2.4398$\;\times \;10^{-1}$ (3.03$\;\times \;10^{-5}$) -	2.4397$\;\times \;10^{-1}$ (3.54$\;\times \;10^{-5}$) -	2.4390$\;\times \;10^{-1}$ (4.64$\;\times \;10^{-8}$) -	**2.4399$\;\times \;10^{-1}$ (1.53$\;\times \;10^{-6}$)**
+/-/=			0/2/3	0/2/3	1/3/1	
**Problem**	**M**	**D**	**NSGAII**	**MOEADDE**	**MMOPSO**	**CMOSCA**
Four bar truss design	2	4	5.9810$\;\times \;10^{-1}$ (2.61$\;\times \;10^{-9}$) -	5.9810$\;\times \;10^{-1}$ (1.06$\;\times \;10^{-7}$) -	5.9810$\;\times \;10^{-1}$ (2.68$\;\times \;10^{-9}$) -	**5.9810$\;\times \;10^{-1}$ (9.02$\;\times \;10^{-10}$)**
Hatch cover design	2	2	8.8747$\;\times \;10^{-1}$ (1.28$\;\times \;10^{-5}$) -	8.8665$\;\times \;10^{-1}$ (4.11$\;\times \;10^{-6}$) -	8.8746$\;\times \;10^{-1}$ (1.98$\;\times \;10^{-5}$) -	8.8750$\;\times \;10^{-1}$ (5.81$\;\times \;10^{-6}$)
Two bar truss design	3	3	9.7451$\;\times \;10^{-1}$ (1.50$\;\times \;10^{-2}$) -	**9.9176$\;\times \;10^{-1}$ (6.66$\;\times \;10^{-5}$) =**	9.7852$\;\times \;10^{-1}$ (1.02$\;\times \;10^{-2}$) -	9.8546$\;\times \;10^{-1}$ (7.64$\;\times \;10^{-3}$)
Welded beam design	3	4	8.9927$\;\times \;10^{-1}$ (3.05$\;\times \;10^{-1}$) +	**9.9991$\;\times \;10^{-1}$ (2.13$\;\times \;10^{-5}$) +**	9.9874$\;\times \;10^{-1}$ (9.26$\;\times \;10^{-4}$) +	6.0661$\;\times \;10^{-1}$ (4.60$\;\times \;10^{-1}$)
Vehicle crashworthiness design	3	5	2.4399$\;\times \;10^{-1}$ (4.79$\;\times \;10^{-7}$) -	2.4399$\;\times \;10^{-1}$ (1.60$\;\times \;10^{-5}$) -	2.4395$\;\times \;10^{-1}$ (4.47$\;\times \;10^{-5}$) -	**2.4399$\;\times \;10^{-1}$ (1.53$\;\times \;10^{-6}$)**
+/-/=			1/4/0	1/3/1	1/4/0	

“+”, “=” and “-” indicate that the results of competing MOEAs are statistically superior, similar, and inferior to results obtained by the CMOSCA, respectively. The best result of each test problem is displayed in bold.

## Data Availability

The data presented in this study are available through email upon request to the corresponding author.

## Abbreviations

**Abbreviation**	**Definition**
CMOPSO	competitive mechanism based multi-objective particle swarm optimizer
CMOSCA	multi-objective sine cosine algorithm based on the competitive mechanism
CMOSCAA	multi-objective sine cosine algorithm based on the angle competitive mechanism
CMOSCAD	multi-objective sine cosine algorithm based on the Euclidean distance competitive mechanism
CSO	competitive swarm optimizer
DE	differential evolution
DTLZ	scalable test problems for evolutionary multiobjective optimization, DTLZ are abbreviations of the reference authors
EA-M2SCA	energy-aware multi-objective modified sine cosine algorithm
EA-MHSCA	hybrid improved version multi-objective modified sine cosine algorithm
EED	environmental/economic dispatch
EELD	economic emission load dispatch
EMOSO	efficient multi-objective optimization algorithm based on level swarm optimizer
FES	current number of function evaluations
HV	hypervolume
IBEA	indicator-based evolutionary algorithm
MaOSCA	many-objective sine cosine algorithm
MaxFES	maximum number of function evaluations
MMOPSO	multi-objective particle swarm optimization with multiple search strategies
MOEA/D	multiobjective evolutionary algorithm based on decomposition
MOEA/D-AM2M	a new variant of MOEA/D-M2M with adaptive adjustment
MOEA/DD	evolutionary many-objective optimization algorithm based on dominance and decomposition
MOEA/D-DE	MOEA/D with differential evolution operators
MOEA/D-M2M	decomposition of a multiobjective optimization problem into a number of simple multiobjective subproblems
MOEA/D-PaS	decomposition-based algorithms using pareto adaptive scalarizing methods
MOEAs	multi-objective evolutionary algorithms
MOPs	multi-objective optimization problems
MOSCA	multi-objective sine cosine algorithm
MOSCA-SSC	multi-objective sine cosine algorithm for spatial-spectral clustering
MPS	multiprocessor systems
MSCA	modified sine cosine algorithm
MSCO	multi-objective sine cosine optimization algorithm
NSGA-II	fast and elitist multiobjective genetic algorithm
NSGA-III	evolutionary many-objective optimization algorithm using reference-point-based nondominated sorting approach
PF	Pareto front
PlatEMO	platform for evolutionary multi-objective optimization
PS	Pareto-optimal set
PSO	particle swarm optimization
RVEA	reference vector guided evolutionary algorithm
SCA	sine cosine algorithm
SDE	shift-based density estimation
SMS-EMOA	s-metric selection based MOEA
TC-MOPSO	multiobjective particle swarm optimization algorithm based on tripartite competition mechanism
Two-arch2	improved two-archive algorithm for many-objective optimization
VEGA	vector evaluated genetic algorithm
WFG	scalable multi-objective test problem toolkit, WFG is abbreviation of Walking Fish Group
ZDT	multi-objective test problem, ZDT are abbreviations of the reference authors
